# Supplementary material for: Unified and explainable molecular representation learning for imperfectly annotated data from the hypergraph view
Source: Nat Commun. 2025 Sep 30;16:8717. doi: 10.1038/s41467-025-63730-6 (PMC12484882; doi:10.1038/s41467-025-63730-6)
Supplement: Supplementary file 1 — Supplementary Information [file 41467_2025_63730_MOESM1_ESM.pdf]

Unified and Explainable Molecular Representation Learning for Imperfectly Annotated Data from the Hypergraph View

Bowen Wang[1,†], Junyou Li[2,†], Donghao Zhou[1], Lanqing Li[1,2], Jinpeng Li[1], Ercheng Wang[2], Jianye Hao[3,*], Liang Shi[4], Chengqiang Lu[5], Jiezhong Qiu[2], Tingjun Hou[6,*], Dongsheng Cao[7,*], Guangyong Chen[8,*], Pheng Ann Heng[1]

[1]Department of Computer Science and Engineering, The Chinese University of Hong Kong, Hong Kong, China
[2]Zhejiang Lab, Hangzhou, China
[3]College of Intelligence and Computing, Tianjin University, Tianjin, China
[4]Chemistry and Biochemistry, University of California, Merced, California, United States
[5]University of Science and Technology of China, Hefei, China
[6]Zhejiang University, Hangzhou, China
[7]Xiangya School of Pharmaceutical Sciences, Central South University, Changsha, China
[8]Hangzhou Institute of Medicine, Chinese Academy of Science, Hangzhou, China

† These authors contributed equally to this work.
* Corresponding authors: Jianye Hao (jianye.hao@tju.edu.cn), Tingjun Hou (tingjunhou@zju.edu.cn), Dongsheng Cao (oriental-cds@163.com), Guangyong Chen (gychen@link.cuhk.edu.hk)

Table of Contents

1. Comparison with Additional Benchmark Models . . . 2
2. Full Evaluation Metrics and Standard Deviations . . . 3
3. Effectiveness of Proposed Modules . . . 3
4. Essential Factors Influencing Molecule ADMET-P Properties . . . 6
5. Readout Block Computational Details . . . 6
6. Demonstrating Chirality Awareness in the SE(3)-Encoder . . . 7
7. Abbreviation and Explanation . . . 9
8. Dataset Overview . . . 16
9. Training Configurations . . . 17
10. Similarity Info of Dataset . . . 17
11. Physiochemical Descriptors of Dataset . . . 19
12. Additional Evaluation with Alignment Operations . . . 21
13. Benchmarking Against Traditional ADMET Prediction Methods . . . 21
14. Statistical Performance Comparison with Traditional Methods . . . 23
15. Analysis of Meta-Embedding Evolution Through Training . . . 25
16. Statistical Validation Through Y-Randomization Tests . . . 25
17. Data Aligned Comparison with HelixADMET Using Scaffold Split . . . 26
18. Comparative Analysis of Atomic Attention Patterns Across ADMET Endpoints . . . 28
19. Comparative Validation of Structure-Activity Relationship Interpretations . . . 29
20. Applicability Domain Analysis . . . 30
21. Effectiveness of the Geometry-Related Modules . . . 34
22. Reproducibility Example for LogS Prediction . . . 37

1 Comparison with Additional Benchmark Models

Supplementary Table 1 Performance Comparison with Benchmark Models (ROC-AUC). The results for HelixADMET and admetSAR 2.0 reflect the overlapped ADMET-P properties.

Names	admetSAR 2.0	HelixADMET	ADMETlab 2.0	OmniMol
Pgp-inh	0.931	0.940	0.922	0.942
Pgp-sub	0.865	0.860	0.840	0.907
HIA	-	-	0.866	0.940
$F_{20\%}$	-	-	0.833	0.933
$F_{30\%}$	-	-	0.848	0.910
BBBP	0.944	0.944	0.908	0.922
CYP1A2-inh	0.883	0.948	0.928	0.934
CYP1A2-sub	-	0.926	0.737	0.976
CYP2C19-inh	0.871	0.938	0.913	0.924
CYP2C19-sub	-	0.934	0.758	0.958
CYP2C9-inh	0.858	0.923	0.919	0.919
CYP2C9-sub	0.625	0.944	0.725	0.902
CYP2D6-inh	0.840	0.904	0.892	0.917
CYP2D6-sub	0.772	0.948	0.847	0.903
CYP3A4-inh	0.848	0.929	0.921	0.922
CYP3A4-sub	0.695	0.956	0.776	0.810
$T_{1/2}$	-	-	0.801	0.851
hERG inhibition	0.811	0.906	0.943	0.942
Hepatotoxicity	0.719	0.808	0.814	0.794
DILI	-	-	0.924	0.932
Ames mutagenicity	0.914	0.898	0.902	0.907
Rodent Acute Toxicity	-	0.562	0.853	0.835
FDAMDD	-	-	0.804	0.837
SkinSen	-	-	0.707	0.842
Carcinogenicity	0.847	0.807	0.788	0.806
EC	-	-	0.983	0.996
EI	-	-	0.982	0.982
Respiratory	-	-	0.828	0.876
NR-AR	0.886	0.858	0.886	0.931
NR-AR-LBD	-	-	0.915	0.934
NR-AhR	-	0.919	0.943	0.952
NR-Aromatase	0.886	0.886	0.852	0.884
NR-ER	0.880	0.848	0.771	0.837
NR-ER-LBD	-	-	0.850	0.915
NR-PPAR-γ	0.818	0.861	0.893	0.896
SR-ARE	-	0.884	0.863	0.888
SR-ATAD5	-	0.921	0.874	0.867
SR-HSE	-	0.884	0.907	0.912
SR-MMP	-	0.951	0.927	0.953
SR-p53	-	0.935	0.881	0.911
Average	-	-	0.863	0.905
Compare with HelixADMET	-	0.894	-	0.902
Compare with admetSAR 2.0	0.836	-	-	0.895

Supplementary Table 1 outlines the performance comparison between OmniMol and other leading models for ADMET-P prediction, namely admetSAR 2.0, HelixADMET, and ADMETlab 2.0. It is important to note the discrepancies in dataset sizes between these models and OmniMol, particularly the datasets used by HelixADMET, which are substantially larger than ours. This includes the cytochrome P450 substrate datasets, where HelixADMET demonstrates superior performance due to the larger dataset size. Additionally, the target sets for ADMET-P prediction between our model and others are not directly comparable. Despite these challenges, we provide a fair comparison by reporting the average Area Under the Curve (AUC) for the ADMET-P prediction tasks that overlap between the models. Our findings reveal that OmniMol surpasses previous methods in both the diversity of ADMET-P prediction targets and achieving higher average Receiver Operating Characteristic (ROC-AUC) performance.

2 Full evaluation metrics and standard deviations

In addition to the primary metrics discussed in the main text, we extend our analysis through a comprehensive evaluation utilizing a range of supplementary performance indicators. For each molecule in our dataset, we have generated 11 distinct conformations at random. We conducted multiple inference experiments, specifically repeating the experiment denoted by model **D** in Supplementary Table 4 five times. In each iteration, a different random set of conformations was selected. This approach allows us to assess the robustness of our method across various generated conformations. Furthermore, the expanded set of evaluation metrics furnishes a more thorough understanding of our results. We present statistical results after five iterations. For classification tasks, the metrics reported include accuracy, ROC AUC, specificity, sensitivity, and the Matthews Correlation Coefficient (MCC). For regression tasks, we include R^2, mean absolute error (MAE), root mean square error (RMSE), concordance correlation coefficient (CCC), and several validation metrics (Q^2_{F1}, Q^2_{F2}, Q^2_{F3}, and r^2_m). For each metric, both the mean value and the standard deviation are reported to illustrate the stability of the model's performance.

3 Effectiveness of proposed modules

As presented in Supplementary Table 4, our research comprehensively examines the efficacy of various components within the proposed model. The ADMETLab 2.0 column provides a reference for the averaged performance across all metrics, serving as our baseline method. To evaluate the effectiveness of different methodological ideas, we compared six variants of our model:

- DRFormer: The use of iterative geometry update with the DR-Label strategy.
- SE(3)-Encoder: Incorporation of an SE(3)-encoder to enhance chirality recognition capabilities.
- Meta Encoder: Utilization of meta-information from diverse tasks to augment the task-routed mixture of expert modules.
- Fine-tune (FT): Refinement of the model through fine-tuning on each specific endpoint from a pre-trained model.
- Mixed training: Whether the model undergoes training across 52 different ADMET-P tasks or is used as a task-specific model for single property prediction.

Our findings are as follows:

1. Model **D**, which deployed all methodological modules, significantly outperformed ADMETLab 2.0, especially in regression tasks, without fine-tuning on each endpoint. This enhancement was further amplified through **D-FT**, achieving the best performance by fine-tuning the pre-trained model **D**.
2. To evaluate the importance of mixed training, experiment **A** deployed the full OmniMol architecture but was trained on each dataset individually. Compared to **D**, **A** showed inferior performance. The mixed training approach in **D** resulted in a 21.3% relative increase in average ROC-AUC for classification tasks and a 57.8% relative decrease in average MAE for regression tasks. This suggests that increasing training samples bolsters model performance.
3. To assess the effectiveness of the "SE(3)-Encoder" and "Meta Encoder" modules, we conducted experiments **B** (both modules removed) and **C** (only meta encoder removed). Comparing **B** and **C**, we observed notable improvements in average performance on both classification and regression tasks. This differentiation is further revealed in our chirality-awareness experiments incorporating the SE(3)-encoder, underscoring its necessity and efficacy in enabling the model to effectively differentiate between enantiomers with distinct ADMET-P properties.

Supplementary Table 2 Model performance on classification datasets

Dataset	Accuracy		ROC AUC		Specificity		Sensitivity		MCC	
	mean	STD	mean	STD	mean	STD	mean	STD	mean	STD
Ames	0.835	0.004	0.908	0.001	0.821	0.009	0.847	0.003	0.667	0.009
BBBP	0.842	0.009	0.924	0.002	0.784	0.010	0.895	0.012	0.685	0.018
CYP1A2-inh	0.865	0.003	0.928	0.001	0.883	0.005	0.844	0.003	0.729	0.007
CYP1A2-sub	0.805	0.023	0.876	0.008	0.758	0.029	0.856	0.030	0.616	0.046
CYP2C19-inh	0.855	0.005	0.923	0.002	0.869	0.005	0.837	0.004	0.707	0.009
CYP2C19-sub	0.769	0.038	0.888	0.009	0.680	0.056	0.891	0.041	0.569	0.070
CYP2C9-inh	0.857	0.003	0.917	0.001	0.896	0.005	0.778	0.004	0.676	0.007
CYP2C9-sub	0.807	0.005	0.855	0.010	0.853	0.009	0.739	0.017	0.597	0.012
CYP2D6-inh	0.890	0.003	0.887	0.003	0.943	0.003	0.672	0.006	0.638	0.010
CYP2D6-sub	0.836	0.006	0.896	0.004	0.787	0.020	0.886	0.016	0.676	0.011
CYP3A4-inh	0.841	0.002	0.911	0.001	0.870	0.004	0.801	0.002	0.672	0.005
CYP3A4-sub	0.739	0.013	0.746	0.008	0.692	0.011	0.784	0.024	0.479	0.027
Carcinogenicity	0.729	0.017	0.780	0.009	0.660	0.023	0.800	0.016	0.464	0.034
DILI	0.877	0.018	0.916	0.008	0.904	0.019	0.850	0.023	0.755	0.035
EC	0.974	0.000	0.995	0.000	0.979	0.000	0.966	0.000	0.945	0.000
EI	0.954	0.004	0.977	0.001	0.910	0.016	0.969	0.002	0.880	0.010
F(20%)	0.830	0.007	0.913	0.007	0.704	0.022	0.872	0.007	0.561	0.019
F(30%)	0.832	0.012	0.900	0.007	0.794	0.000	0.851	0.018	0.633	0.023
FDAMDD	0.744	0.013	0.812	0.005	0.778	0.020	0.705	0.015	0.485	0.026
H-HT	0.698	0.005	0.764	0.004	0.690	0.009	0.704	0.008	0.391	0.011
HIA	0.917	0.007	0.868	0.005	0.800	0.000	0.934	0.008	0.669	0.019
NR-AR	0.981	0.002	0.874	0.006	0.992	0.002	0.662	0.017	0.700	0.031
NR-AR-LBD	0.982	0.002	0.958	0.002	0.994	0.001	0.626	0.050	0.695	0.036
NR-AhR	0.931	0.004	0.929	0.003	0.961	0.003	0.699	0.011	0.660	0.016
NR-Aromatase	0.945	0.001	0.851	0.007	0.973	0.002	0.331	0.021	0.314	0.015
NR-ER	0.903	0.004	0.812	0.003	0.957	0.004	0.454	0.017	0.451	0.018
NR-ER-LBD	0.963	0.001	0.815	0.006	0.990	0.001	0.435	0.025	0.526	0.014
NR-PPAR-γ	0.977	0.002	0.804	0.016	0.993	0.002	0.460	0.022	0.548	0.033
Pgp-inh	0.882	0.004	0.924	0.002	0.880	0.009	0.883	0.003	0.757	0.009
Pgp-sub	0.805	0.012	0.882	0.004	0.784	0.014	0.825	0.017	0.609	0.024
ROA	0.774	0.006	0.838	0.002	0.813	0.008	0.710	0.009	0.522	0.012
Respiratory	0.799	0.006	0.867	0.006	0.779	0.010	0.812	0.005	0.585	0.013
SR-ARE	0.891	0.003	0.868	0.004	0.953	0.005	0.554	0.010	0.552	0.009
SR-ATAD5	0.961	0.002	0.865	0.004	0.987	0.001	0.240	0.040	0.286	0.040
SR-HSE	0.954	0.005	0.889	0.012	0.980	0.003	0.511	0.058	0.534	0.051
SR-MMP	0.934	0.002	0.941	0.002	0.967	0.003	0.752	0.017	0.738	0.009
SR-p53	0.951	0.002	0.877	0.007	0.984	0.002	0.494	0.023	0.559	0.022
SkinSen	0.675	0.018	0.671	0.020	0.477	0.034	0.770	0.017	0.250	0.041
$T_{1/2}$	0.715	0.025	0.838	0.005	0.784	0.019	0.608	0.056	0.397	0.056
hERG	0.882	0.003	0.938	0.000	0.868	0.002	0.896	0.006	0.764	0.007

Supplementary Table 3 Model performance on regression datasets

Dataset	R^2		MAE		RMSE		CCC		Q^2_{F1}		Q^2_{F2}		Q^2_{F3}		r^2_m	
	mean	STD	mean	STD	mean	STD	mean	STD	mean	STD	mean	STD	mean	STD	mean	STD
BCF	0.767	0.005	0.472	0.005	0.634	0.006	0.869	0.003	0.779	0.004	0.763	0.005	0.785	0.004	0.753	0.005
Caco-2	0.868	0.005	0.204	0.005	0.294	0.006	0.927	0.003	0.868	0.005	0.866	0.005	0.879	0.005	0.865	0.006
Fu	0.808	0.003	0.216	0.001	0.331	0.003	0.894	0.002	0.808	0.003	0.807	0.003	0.789	0.004	0.794	0.005
IGC_{50}	0.839	0.003	0.239	0.003	0.380	0.004	0.914	0.002	0.839	0.003	0.837	0.003	0.875	0.002	0.810	0.004
LC_{50}	0.758	0.005	0.539	0.004	0.848	0.009	0.867	0.003	0.763	0.005	0.754	0.005	0.669	0.007	0.715	0.007
$LC_{50}DM$	0.653	0.014	0.657	0.013	0.862	0.019	0.789	0.009	0.563	0.019	0.546	0.020	0.766	0.010	0.539	0.018
LogD	0.921	0.001	0.293	0.002	0.397	0.001	0.958	0.000	0.920	0.001	0.920	0.001	0.916	0.001	0.917	0.002
LogP	0.963	0.001	0.223	0.002	0.329	0.003	0.981	0.000	0.963	0.001	0.963	0.001	0.968	0.001	0.955	0.001
LogS	0.875	0.001	0.507	0.003	0.798	0.002	0.935	0.000	0.871	0.001	0.871	0.001	0.866	0.001	0.845	0.001
MDCK	0.768	0.007	0.191	0.002	0.272	0.004	0.859	0.005	0.767	0.007	0.765	0.007	0.752	0.007	0.724	0.007
PPB	0.853	0.000	0.057	0.000	0.101	0.000	0.920	0.000	0.849	0.000	0.849	0.000	0.865	0.000	0.849	0.002
VD_{ss}	0.779	0.008	0.364	0.006	0.728	0.015	0.880	0.005	0.743	0.011	0.742	0.011	0.770	0.010	0.675	0.010

4. Comparing **C** and **D**, we observed that while classification task performance remained on par, adding the meta-encoder provided effective improvement in regression task performance. This indicates that the meta-information encoder can

Supplementary Table 4 Evaluation of the effectiveness of crucial components introduced by OmniMol. Top result in **bold**

			Models						
			ADMETLab 2.0	Ours					
				A	B	C	C+FT	D	D+FT
Modules		DRFormer	×	√	√	√	√	√	√
		SE(3)-Encoder	×	√	×	√	√	√	√
		Meta Encoder	×	√	×	×	×	√	√
		Mixed Training	√	×	√	√	√	√	√
		Fine Tune	√	×	×	×	√	×	√
Metric	Classification	ROC-AUC	0.863	0.715	0.848	0.867	0.881	0.867	**0.894**
		ACC	0.824	0.727	0.827	0.857	0.876	0.848	**0.881**
	Regression	R^2	0.770	0.226	0.673	0.796	0.829	0.818	**0.839**
		MAE	0.377	0.800	0.469	0.350	0.327	0.338	**0.319**

effectively allocate different experts based on targeted downstream tasks, increasing model versatility and adaptability.

5. Fine-tuned results in **C-FT** and **D-FT** demonstrated that mixed trained models have further potential for achieving better performance with model fine-tuning. Notably, fine-tuning a mixed pre-trained model demands equivalent computational resources as training from scratch (**A**). However, comparing **A**, **C-FT**, and **D-FT**, the drastically enhanced performance shows the effectiveness of a mixed pre-trained model.

These findings indicate that understanding inter-task relationships can be instrumental in enabling models to acquire comprehensive druggability knowledge across various tasks. The SE(3)-encoder's ability to differentiate enantiomers is particularly important in drug discovery, where molecular chirality plays a crucial role and represents a large proportion of the datasets.

4 Essential Factors Influencing Molecule ADMET-P properties

Supplementary Table 5 offers an exhaustive review of critical factors and structural alerts pivotal for various ADMET-P tasks. It provides a foundation for interpreting results, highlighting the significant functional groups that OmniMol identifies as having increased attention values.

Supplementary Table 5 Comprehensive Overview of Factors Influencing Molecule ADMET-P

Task	Key Factors and Structural Alerts	Ref.
Ames	Aromatic amines, Amides, Hydroxylamines, Azo compounds, Aromatic Amine, Nitro, Nitroso, Aliphatic halide(excluding the fluorine atom), Polycyclic aromatic system, Acyl halide, Alkyl esters of either phosphonic or sulphonic acids, Aromatic N-oxides, Hydrazines, Aldehydes, S- or N-mustards, Epoxides and aziridines, Halogenated benzene, etc.	[1, 12, 17, 25]
Carcinogenicity	Alkyl esters of either phosphonic or sulphonic acids, Aromatic nitro or nitroso groups, Aromatic N-oxides, Aromatic amines, hydrazines, aldehydes S- or N-mustards, Acyl halides, epoxides and aziridines, Aliphatic halogens, Aromatic hydroxylamine, Hydrazo derivatives, Aliphatic halides,Benzylic halide, etc.	[1, 25]
CYP450s-inh	Nitrogen-containing heteroaromatic molecules, Such as pyridine, quinoline, imidazole and triazole derivatives, Terminal acetylenes, Olefins, Furans and thiophenes, epoxides, amines, dichloro- and Trichloroethylenes, Methylenedioxyphenyl, Conjugated structures, Hydrazines, Isothiocyanates, Thioamides, Dithiocarbamates, Michael acceptors, etc.	[16]
H-HT or DILI	Sulfonamides, Anilines, Furan, Alkylphenols, Hydrazines Halogens, Phenylpropanoids, Polyketides, Organic acids, Double bonds, Ketones, Halogens, Sulfur, Phosphorus, Aromatic nitro, Nitroso and amines, Oxime, Epoxide, Aziridine, Azide, Diazo, Aliphatic halide, Sulphur mustard, Nitrogen mustard, Hydrazine, Acyl halide, etc.	[12, 15, 21]
$F_{20\%}/F_{30\%}$	Molecular flexibility (rotatable bond), Polar surface area, Hydrogen bond (acceptor and donor), Molecular weight, Lipophilicity, etc.	[32]
hERG	Formation of zwitterions, Lipophicity, PKa, $\pi - \pi$ stacking altering, Molecular rigidity, Polarity, Quaternary ammonium salt, Tertiary amine, Basic nitrogen atoms, etc.	[9, 30]
HIA	Number of H-bond, Molecular flexibility, Total positive polar van der Waals surface area, Solvation energy, Existence of positively charged N atom, Number of violations of rule-of-five rules, etc.	[34]
Pgp-sub	Molecular weight >400, Number of N+O >8, PKa >4, etc.	[14]
Respiratory	Thiazol-2-amine, Guanidine, Isocyanic acid, Thiol derivatives, Pyrimidin-4-amine, 2-aminoethyl formate, Ethanolamine derivatives, 2-phenoxyethan-1-amine, etc.	[36]
BBBP	Molecular weight, LogP, LogD, TPSA, HBD, PKa, Amines, Carboxylic acid, Hydroxyl, etc.	[10]
Skin sensibility	Hydrazines, Hydrazonium salts and precursors, Acid halides, Aldehydes, Halonitrobenzenes, Aromatic sulphinic acids and salts, Acid anhydrides, Iso(thio)cyanates, (Benzo)isothiazolin-ones, Epoxides, Acid imides, Diamines,etc.	[11]
EC/EI	Acyl chlorides, Aldehydes, Active bromine atoms, Oximes, Nitrosos, Aromatic carboxylic acids, Aromatic amines, Cyano groups, Sulphides, Oxidizing groups, etc.	[6, 35]
PPB	Nitrogenous groups, Lipophicity, Pka, etc	[22]
ROA	Sulphides, Phosphate esters, Nitriles, Alkyl halides, Nitro groups, Nitrosos, Aldehydes, Aromatic amines, Quinones,etc	[23]

5 Readout Block Computational Details

The operational intricacies of the readout block within the OmniMol framework are illustrated in Supplementary Fig. 1. The figure's left section outlines the geometry supervision component of OmniMol. It describes how the node-wise loss is determined by penalizing the discrepancy between the predicted positions and the labeled positions from the previous iteration (N-1). Concurrently, the projection magnitude, derived

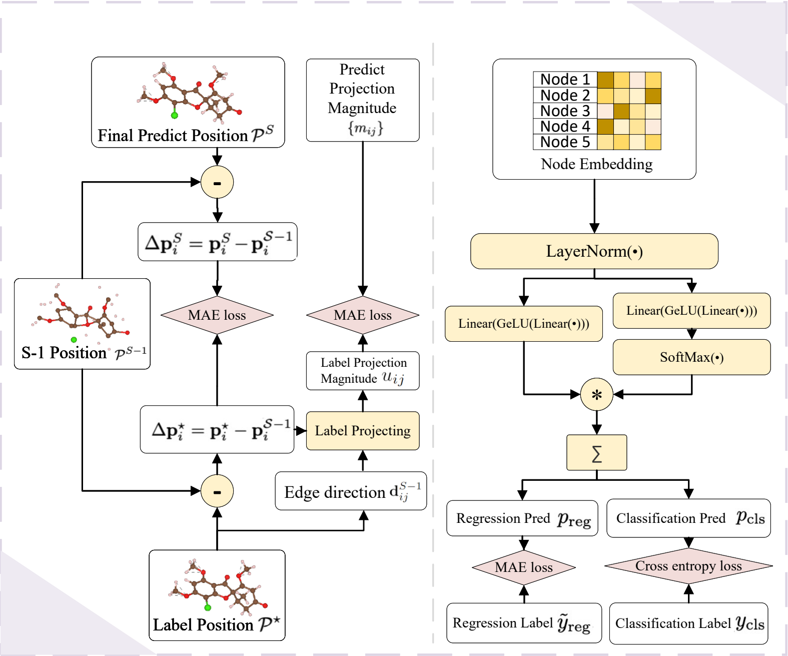

Supplementary Fig. 1 **Detailed decomposition of the readout block.** The left panel illustrates geometry supervision for the final update step (S), where the predicted update vector $\Delta \mathbf{p}_i^S$ (from positions $\mathbf{P}^{S-1}$ to final positions $\mathbf{p}_i^S$) and the projection magnitude m_{ij} are supervised using label projection magnitude u_{ij}. The right panel shows the property prediction heads, where Σ denotes the summation over nodes. For each molecule, it generate a regression value p_{reg} or a classification probability p_{cls}. These are compared against the normalized ground truth label $\tilde{y}_{\text{reg}}$ and the classification label y_{cls}, respectively.

from the preceding positional update, undergoes supervision through the calculation of the Mean Absolute Error (MAE) loss. This MAE loss is assessed between the projection magnitude, which is computed by projecting the label's delta position onto each edge direction.

On the right, the process for generating property predictions is detailed. Following the derivation of the final node-level embeddings, two separate pathways of feed-forward networks are combined with one path incorporating a Softmax function to produce graph-level representations. For regression tasks, the MAE loss between the predicted results and the regression labels is calculated. In contrast, classification tasks utilize the cross-entropy loss to determine the discrepancy between predictions and actual labels.

6 Demonstrating Chirality Awareness in the SE(3)-Encoder

Building upon the calculations, the generated chirality-aware representation $\mathbf{CH}_l^{(\omega)}(\psi)$ will is sensitive to geometric reflections. In this section, we make formal proof to such statement.

Let $\mathbf{f}_{ref}$ be the reflection operation of a molecular system. Two crucial property of cross products and dot product under reflection (mirrored by some plane) is:

$\mathbf{f}_{ref}(a) \times \mathbf{f}_{ref}(b) = -\mathbf{f}_{ref}(a \times b)$

$\langle \mathbf{f}_{ref}(a), \mathbf{f}_{ref}(b) \rangle = \langle a, b \rangle$

where a and b are two vectors.

Therefore, under the reflection on the molecular system, we have that

$$\begin{aligned}
\tilde{\mathbf{c}}_{ij} &= \sum_{k \in \mathcal{N}_i \backslash j} \mathbf{f}_{ref}(\mathbf{d}_{ij}) \times \mathbf{f}_{ref}(\mathbf{d}_{ik}) && (1)\\
&= \sum_{k \in \mathcal{N}_i \backslash j} -\mathbf{f}_{ref}(\mathbf{d}_{ij} \times \mathbf{d}_{ik}) && (2)\\
&= -\mathbf{f}_{ref}(\sum_{k \in \mathcal{N}_i \backslash j} \mathbf{d}_{ij} \times \mathbf{d}_{ik}) && (3)\\
&= -\mathbf{f}_{ref}(\mathbf{c}_{ij}) && (4)
\end{aligned}$$

For the same way, $\tilde{\mathbf{c}}_{ij} = -\mathbf{f}_{ref}(\mathbf{c}_{ij})$

Next, since

$$\begin{aligned}
\left\langle \frac{\tilde{\mathbf{c}}_{ij} \times \tilde{\mathbf{c}}_{ji}}{|\tilde{\mathbf{c}}_{ij}| \cdot |\tilde{\mathbf{c}}_{ji}|}, \tilde{d}_{ij} \right\rangle &= \left\langle \frac{-\mathbf{f}_{ref}(\mathbf{c}_{ij}) \times -\mathbf{f}_{ref}(\mathbf{c}_{ji})}{|-\mathbf{f}_{ref}(\mathbf{c}_{ij})| \cdot |-\mathbf{f}_{ref}(\mathbf{c}_{ji})|}, \mathbf{f}_{ref}(\mathbf{d}_{ij}) \right\rangle && (5)\\
&= \left\langle \frac{-\mathbf{f}_{ref}(\mathbf{c}_{ij} \times \mathbf{c}_{ji})}{|\mathbf{c}_{ij}| \cdot |\mathbf{c}_{ji}|}, \mathbf{f}_{ref}(\mathbf{d}_{ij}) \right\rangle && (6)\\
&= -\left\langle \mathbf{f}_{ref}(\frac{\mathbf{c}_{ij} \times \mathbf{c}_{ji}}{|\mathbf{c}_{ij}| \cdot |\mathbf{c}_{ji}|}), \mathbf{f}_{ref}(\mathbf{d}_{ij}) \right\rangle && (7)\\
&= -\left\langle \frac{\mathbf{c}_{ij} \times \mathbf{c}_{ji}}{|\mathbf{c}_{ij}| \cdot |\mathbf{c}_{ji}|}, \mathbf{d}_{ij} \right\rangle, && (8)
\end{aligned}$$

Therefore, since

$$\psi_{ij} = \text{asin}\left(\left\langle \frac{\mathbf{c}_{ij} \times \mathbf{c}_{ji}}{|\mathbf{c}_{ij}| \cdot |\mathbf{c}_{ji}|}, \mathbf{d}_{ij} \right\rangle \right). \quad (9)$$

let $\tilde{\psi_{ij}} = \text{asin}\left(\left\langle \frac{\tilde{\mathbf{c}}_{ij} \times \tilde{\mathbf{c}}_{ji}}{|\tilde{\mathbf{c}}_{ij}| \cdot |\tilde{\mathbf{c}}_{ji}|}, \tilde{d}_{ij} \right\rangle \right)$ be the corresponding torsion angle of ψ_{ij} under reflection, we have

$$\begin{aligned}
\tilde{\psi_{ij}} &= \text{asin}\left(-\left\langle \mathbf{f}_{ref}(\frac{\mathbf{c}_{ij} \times \mathbf{c}_{ji}}{|\mathbf{c}_{ij}| \cdot |\mathbf{c}_{ji}|}), \mathbf{f}_{ref}(\mathbf{d}_{ij}) \right\rangle \right) && (10)\\
&= -\text{asin}\left(\left\langle \mathbf{f}_{ref}(\frac{\mathbf{c}_{ij} \times \mathbf{c}_{ji}}{|\mathbf{c}_{ij}| \cdot |\mathbf{c}_{ji}|}), \mathbf{f}_{ref}(\mathbf{d}_{ij}) \right\rangle \right) && (11)\\
&= -\psi_{ij} && (12)
\end{aligned}$$

Therefore, when $l = 1$

$$\mathbf{CH}_l^{(\omega)}(\tilde{\psi_{ij}}) = \sin(\omega \tilde{\psi_{ij}}) = \sin(-\omega \psi_{ij}) = -\sin(\omega \psi_{ij}) = -\mathbf{CH}_l^{(\omega)}(\psi_{ij}) \quad (13)$$

The representation of corresponding torsion angle will be negated based on our method.

Node permutation invariance is achieved by summing over all neighboring nodes, where the addition operation is by itself permutation invariant.

7 Abbreviation and Explanation

Supplementary Table 6: Abbreviations and Explanations of all ADMET-P endpoints in OmniMol

Endpoint name	Endpoint abbreviation	Endpoint explanation
		**Absorption**
P-glycoprotein inhibitor	Pgp-inh	P-glycoprotein belongs to the superfamily of ATP-binding cassette (ABC) transporters. A compound inhibiting p-glycoprotein can block its ability to transport other compounds out of the cell, leading to an increased intracelluar concentration of these compounds. The output value is the probability of being a pgp-inhibitor, within the range of 0 to 1 .
P-glycoprotein substrate	Pgp-sub	If a compound is a P-glycoprotein substrate, it is readily exported from the cell, resulting in a lower intracellular concentration. The output value is the probability of being pgp-substrate, within the range of 0 to 1 .
Human Intestinal Absorption	HIA	The human intestinal absorption of an oral drug is crucial for its perceived effectiveness. Additionally, there is a strong link between oral bioavailability. The output value is the probability of being HIA+, within the range of 0 to 1 .
Human oral bioavailability 20%	F20%	Predicting whether a drug can achieve a human oral bioavailability of 20% or higher.
Human oral bioavailability 30%	F30%	Predicting whether a drug can achieve a human oral bioavailability of 30% or higher.
Human colon adenocarcinoma cell lines permeability	Caco-2	The human colon adenocarcinoma cell line (Caco-2) is often used as an alternative model for human intestinal epithelium, owing to its morphological and functional similarities, to estimate in vivo drug permeability. A compound is considered to have a high Caco-2 permeability if it has predicted value >-5.15log cm/s.
Madin-Darby Canine Kidney cells permeability	MDCK	Madin-Darby Canine Kidney cells (MDCK) have been developed as an in vitro model for permeability screening and ss widely considered to be the in vitro gold standard for assessing the uptake efficiency of chemicals into the body. A compound is considered to have a high MDCK permeability if it has predicted value 20 x 10^{-6} cm/s.

Continued on next page

Supplementary Table 6 – continued from previous page

**Endpoint name**	**Endpoint abbreviation**	**Endpoint explanation**
		**Distribution**
Blood-Brain-Barrier Penetration	BBBP	BBBP assesses a compound's ability to enter the brain. While crossing the blood-brain barrier is crucial for drugs targeting the central nervous system (CNS), it can also cause unwanted side effects. For drugs aimed at peripheral targets, limited or no BBB penetration may be preferable to minimize CNS effects. The output value is the probability of being BBB+, within the range of 0 to 1 .
Plasma Protein Binding	PPB	PPB refers to the percentage of a compound that attaches to the plasm proteins. A drug with higner PPB implies a lower free/unbound concentration. Only the free/unbound drug concentration is responsible for the pharmacological activity. A compound is considered to have a proper PPB if it has predicted value $< 90\%$.
Volume Distribution	VD	The VD is a theoretical concept that connects the administered dose with the actual initial concentration present in the circulation and it is an important parameter to describe the in vivo distribution for drugs. A compound is considered to have a proper VD if it has predicted value in the range of 0.04-20L/kg.
The fraction unbound in plasms	Fu	The fraction unbound in plasms. Most drugs in plasma will exist in equilibrium between either an unbound state or bound to serum proteins. Only the free/unbound drug concentration is responsible for the pharmacological activity.

Continued on next page

Supplementary Table 6 – continued from previous page

**Endpoint name**	**Endpoint abbreviation**	**Endpoint explanation**
		**Excretion**
Half-life time	T1/2	The half-life of a drug refers to the time required for the concentration of the drug in the body to decrease by half. It often involves clearance and volume of distribution.The output value is the probability of a drug has long hale-life (T1/2>3h) within the range of 0 to 1 .
		**Metabolism**
CYP1A2-inhibitor	CYP1A2-inh	In the metabolic phase within the body, drugs are divided into Phase I metabolism reactions (reduction, oxidation, hydrolysis, etc.) and Phase II metabolism reactions (conjugation, plucuronidation, acetylation, sulfation, etc.). The human cytochrome P450 family (phase I enzymes) contains 57 isozymes and these isozymes metabolize approximately two-thirds of known drugs in human with 80% of this attribute to five isozymes—-CYP1A2, CYP2C19, CYP2C9, CYP2D6, CYP3A4. If a compound is the inhibitor of CYPs, it may increase the concentration of other drugs, result in drug accumulation and drug-drug interaction (DDI)-mediated toxicity. If a compound is the substrate of CYPs, it might be oxidized by the enzymes, result in inactive and/or toxic products. The output value is the probability of being substrate / inhibitor, within the range of 0 to 1.
CYP1A2-substrate	CYP1A2-sub	
CYP2C19-inhibitor	CYP2C19-inh	
CYP2C19-substrate	CYP2C19-sub	
CYP2C9-inhibitor	CYP2C9-inh	
CYP2C9-substrate	CYP2C9-sub	
CYP2D6-inhibitor	CYP2D6-inh	
CYP2D6-substrate	CYP2D6-sub	
CYP3A4-inhibitor	CYP3A4-inh	
CYP3A4-substrate	CYP3A4-sub	

Continued on next page

Supplementary Table 6 – continued from previous page

Endpoint name	Endpoint abbreviation	Endpoint explanation
		**Toxicity**
hERG channel inhibition/blocker	hERG inhibition	During cardiac depolarization and repolarization, a voltage-gated potassium channel encoded by the human ether-a-go-go related gene (hERG) plays a major role in the regulation of the exchange of cardiac action potential and resting potential. The hERG blockade/inhibition may cause long QT syndrome (LQTS), arrhythmia, and Torsade de Pointes (TdP), which lead to palpitations, fainting, or even sudden death. With a boundary of $IC_{50} = 10\mu M$, the output value is the probability of being hERG+, within the range of 0 to 1 .
Human Hepatotoxicity	H-HT	The human hepatotoxicity. Drug induced liver injury is of great concern for patient safety and a major cause for drug withdrawal from the market. The output value is the probability of being toxic, within the range of 0 to 1 .
Drug-Induced Liver Injury	DILI	Drug-induced liver injury (DILI) has become the most common safety problem of drug withdrawal from the market. The output value is the probability of being toxic, within the range of 0 to 1 .
Ames test/toxicity	Ames	The Ames trial is to test for mutagenicity of a drug, which has a close relationship with the carcinogenicity, and it is the most widely used assay for testing the mutagenicity of compounds. The output value is the probability of being toxic, within the range of 0 to 1 .
Rat Oral Acute Toxicity	ROA	Determination of acute toxicity in mammals (e.g. rats or mice) is one of the most important tasks for the safety evaluation of drug candidates. With a boundary of 500 mg/kg, the output value is the probability of being toxic, within the range of 0 to 1 .
FDA Maximum (Recommended) Daily Dose	FDAMDD	FDA Maximum (Recommended) Daily Dose provides an estimate of the toxic dose threshold of chemicals in humans. With a boundary of 0,011 mmol/kg, the output value is the probability of being toxic, within the range of 0 to 1 .
Skin Sensitization	SkinSen	Skin sensitization is a potential adverse effect for dermally applied products. The evaluation of whether a compound, that may encounter the skin, can induce allergic contact dermatitis is an important safety concern. The output value is the probability of being toxic, within the range of 0 to 1 .
Carcinogencity	Carcinogenicity	Drug are defined as carcinogenic if after inhalation, ingestion, dermal application or injection they induce (malignant) tumours, increase their incidence or malignancy, or shorten the time of tumour occurrence. The output value is the probability of being toxic, within the range of 0 to 1 .

Continued on next page

**Endpoint name**	**Endpoint abbreviation**	**Endpoint explanation**
Respiratory Toxicity	Respiratory	The respiratory system is relevant in toxicology as a target of toxic effects, and also as a major route of absorption of inhaled gases and atmospheric particles. If inhalation and subsequent absorption reach a toxic threshold, inhaled chemicals have the potential to produce lung disease, The output value is the probability of being toxic, within the range of 0 to 1 .
(Activation/Non-activation of) Nuclear Receptor superfamily Androgen Receptor	NR-AR	Activation of androgen receptor may cause disruption of normal endocrine function as well as interfere with metabolic homeostasis, reproduction, developmental and behavioral functions. The output value is the probability of being AR agonists, within the range of 0 to 1 .
(Activation/Non-activation of) Nuclear Receptor superfamily Androgen Receptor Ligand Binding Domain	NR-AR-LBD	Activation of androgen receptor by binding to ligand binding domain of androgen receptor. The output value is the probability of being actives, within the range of 0 to 1 .
(Activation/Non-activation of) Nuclear Receptor superfamily Aryl hydrocarbon Receptor (AhR)	NR-AhR	Activation of Aryl hydrocarbon Receptor mediates cellular responses to environmental pollutants such as aromatic hydrocarbons through induction of phase I and II enzymes but also interacts with other nuclear receptor signaling pathways. The output value is the probability of being actives, within the range of 0 to 1 .
(Activation/Non-activation of) Nuclear Receptor superfamily Aromatase receptor	NR-Aromatase	Inhibition of Aromatase can catalyzes the conversion of androgen to estrogen and plays a key role in maintaining the androgen and estrogen balance in many of the Endocrine disrupting chemicals-sensitive organs. The output value is the probability of being actives, within the range of 0 to 1 .
(Activation/Non-activation of) Nuclear Receptor superfamily Estrogen receptor	NR-ER	Activation of estrogen receptor may cause disruption of mormal endocrine function. The output value is the probability of being actives within the range of 0 to 1 .
(Activation/Non-activation of) Nuclear Receptor superfamily Estrogen receptor Ligand Binding Domain	NR-ER-LBD	Activation of estrogen receptor by binding to ligand binding domain of androgen receptor may cause disruption of normal endocrine function. The output value is the probability of being actives within the range of 0 to 1 .

Continued on next page

Endpoint name	Endpoint abbreviation	Endpoint explanation
(Activation/Non-activation of) Nuclear Receptor superfamily Peroxisome Proliferator-Activated Receptor Gamma	NR-PPAR−γ	Activation of peroxisome proliferator-activated receptor gamma may alter regulation of glucose and lipid metabolism. The output value is the probability of being actives within the range of 0 to 1 .
(Activation/Non-activation of) Antioxidant Responsive Element	SR-ARE	Antioxidant response element signaling pathway plays an important role in the amelioration of oxidative stress. The output value is the probability of being actives within the range of 0 to 1 .
(Affection/Non-affection of) ATPase Family AAA Domain Containing 5 gene	SR-ATAD5	Activation of ATPase family AAA domain-containing protein 5 may canse various DNA damage. The output value is the probability of being actives within the range of 0 to 1 .
(Activation/Non-activation of) Heat shock Sequence Element	SR-HSE	Activation of heat shock factor response element ay lead to the activation of heat shock response/ unfolded protein response (HSR/UPR). The output value is the probability of being actives within the range of 0 to 1 .
(Change/Non-change of) Mitochondrial Membrane Potential	SR-MMP	Mitochondrial membrane potential is generated by mitochondrial electron transport chain that creates an electrochemical gradient by a series of redox reactions. The output value is the probability of being actives within the range of 0 to 1 .
(Activation/Non-activation of) p53 pathway	SR-p53	Activation of p53 is a good indicator of DNA damage and other cellular stresses. The output value is the probability of being actives within the range of 0 to 1 .
Bioconcentration Factor	BCF	The bioconcentration factor BCF is defined as the ratio of the chemical concentration in biota as a result of absorption via the respiratory surface to that in water at steady state. It is used for considering secondary poisoning potential and assessing risks to human health via the food chain. The unit of BCF is log10(L/kg).
48 hour Tetrahymena pyriformis IGC_{50}	IGC_{50}	48 hours Tetrahymena pyriformis IGC_{50} (concentration of the test chemical in water in mg/L that causes 50% growth inhibition to Tetrahymena pyriformis after 48 hours). The unit of IGC50 is $-\log_{10}\{[\mathrm{mg/L}]/(1000 \cdot \mathrm{MW})\}$.
96 hour fathead minnow LC_{50}	LC_{50}FM	96 hours fathead minnow LC50 (concentration of the test chemical in water in mg/L that causes 50% of fathead minnow to die after 96 hours). The unit of LC50FM is $-\log_{10}\{[\mathrm{mg/L}]/(1000 \cdot \mathrm{MW})\}$.
48 hour Daphnia magna LC_{50}	LC_{50}DM	48 hours Daphnia magna LC50 (concentration of the test chemical in water in mg/L that causes 50% of Daphnia magna to die after 48 hours). The unit of LC50DM is $-\log_{10}\{[\mathrm{mg/L}]/(1000 \cdot \mathrm{MW})\}$.

Continued on next page

Supplementary Table 6 – continued from previous page

**Endpoint name**	**Endpoint abbreviation**	**Endpoint explanation**
Eye Corrosion	EC	Assessing the eye irritation/corrosion (EI/EC) potential of a chemical is a necessary component of risk assessment. Cornea and conjunctiva tissues comprise the anterior surface of the eye, and hence cornea and conjunctiva tissues are directly exposed to the air and easily suffer injury by chemicals. The output value is the probability of being toxic, within the range of 0 to 1 .
Eye Irritation	EI	Same explaination as above
	**Physiochemical property**	
LogS	LogS	The logarithm of aqueous solubility value. The predicted solubility of a compound is given as the logarithm of the molar concentration (log mol/L). Compounds in the range from -4 to 0.5 log mol/L will be considered proper.
LogD7.4	LogD	The logarithm of the n-octanol/water distribution coefficients at pH=7.4. The predicted logD7.4 of a compound is given as the logarithm of the molar concentration (log mol/L). Compounds in the range from 1 to 3 log mol/L will be considered proper.
LogP	LogP	The logarithm of the n-octanol/water distribution coefficient. The predicted logP of a compound is given as the logarithm of the molar concentration (log mol/L). Compounds in the range from 0 to 3 log mol/L will be considered proper.

8 Dataset Overview

This section outlines the datasets utilized in our experiments, detailing splits and associated meta-information. For ADMET-P prediction tasks, datasets were divided into training, validation, and testing sets with an 8:1:1 ratio, while chirality prediction tasks adhered to dataset splits established by prior studies. Meta-information for each dataset includes task types (classification or regression), group categories (e.g., Absorption, Distribution, Metabolism, Excretion, Toxicity, Physiochemical properties, Chirality awareness), and specific statistical metrics such as the positive ratio (t_+ for classification tasks), mean (μ_{reg}), and standard deviation (σ_{reg} for regression tasks).

Supplementary Table 7 Datasets Overview: Incorporates training, validation, and test splits, along with meta-information. "Group" abbreviations denote various task domains, including Absorption (A), Distribution (D), Metabolism (M), Excretion (E), Toxicity (T), Physiochemical properties (P), and Chirality awareness (C). Task types are classified into "Cls" for classification and "Reg" for regression, accompanied by relevant statistical metrics such as positive ratio (t_+), mean (μ_{reg}), and standard deviation (σ_{reg}).

Dataset Name	Abbreviation	Sample				Meta Information				
		Total	Train	Validation	Test	Group	Type	t_+	μ_{reg}	σ_{reg}
Ames Toxicity	Ames	7668	6134	767	767	T	Cls	0.562	-	-
BBB Penetration	BBBP	2039	1631	204	204	D	Cls	0.822	-	-
Bioconcentration Factor	BCF	676	540	68	68	T	Reg	-	1.82	1.37
CYP1A2 inhibitor	CYP1A2-inh	12659	10127	1266	1266	M	Cls	0.464	-	-
CYP1A2 substrate	CYP1A2-sub	371	296	38	37	M	Cls	0.475	-	-
CYP2C19 inhibitor	CYP2C19-inh	12688	10150	1269	1269	M	Cls	0.455	-	-
CYP2C19 substrate	CYP2C19-sub	261	208	27	26	M	Cls	0.409	-	-
CYP2C9 inhibitor	CYP2C9-inh	12136	9708	1214	1214	M	Cls	0.331	-	-
CYP2C9 substrate	CYP2C9-sub	820	656	82	82	M	Cls	0.401	-	-
CYP2D6 inhibitor	CYP2D6-inh	13150	10520	1315	1315	M	Cls	0.194	-	-
CYP2D6 substrate	CYP2D6-sub	885	707	89	89	M	Cls	0.494	-	-
CYP3A4 inhibitor	CYP3A4-inh	12405	9924	1241	1240	M	Cls	0.411	-	-
CYP3A4 substrate	CYP3A4-sub	1007	805	101	101	M	Cls	0.504	-	-
Caco-2 Permeability	Caco-2	1134	954	118	62	A	Reg	-	-5.16	0.85
Carcinogenicity	Carcinogenicity	1042	833	105	104	T	Cls	0.496	-	-
DILI	DILI	472	377	48	47	T	Cls	0.499	-	-
Eye Corrosion	EC	2298	1838	230	230	T	Cls	0.386	-	-
Eye Irritation	EI	5220	4176	522	522	T	Cls	0.742	-	-
$F_{20\%}$	$F_{20\%}$	1006	805	101	100	A	Cls	0.749	-	-
$F_{30\%}$	$F_{30\%}$	1006	805	100	101	A	Cls	0.662	-	-
FDAMDD	FDAMDD	1214	971	122	121	T	Cls	0.473	-	-
Fu	Fu	2604	2083	261	260	D	Reg	-	1.16	0.72
H-HT	H-HT	2395	1916	240	239	T	Cls	0.573	-	-
HIA	HIA	1176	940	118	118	A	Cls	0.874	-	-
IGC_{50}	IGC_{50}	1787	1429	179	179	T	Reg	-	3.27	1.07
LC_{50}	LC_{50}	816	652	82	82	T	Reg	-	4.01	1.47
$LC_{50}DM$	$LC_{50}DM$	347	277	35	35	T	Reg	-	4.78	1.78
Log D	Log D	10376	8300	1038	1038	P	Reg	-	2.25	1.37
Log P	Log P	12696	10156	1270	1270	P	Reg	-	2.01	1.83
Log S	Log S	4801	3840	481	480	P	Reg	-	-2.68	2.18
MDCK Permeability	MDCK	1140	912	114	114	A	Reg	-	-4.79	0.55
NR-AR	NR-AR	7389	5911	740	738	T	Cls	0.034	-	-
NR-AR-LBD	NR-AR-LBD	6927	5542	693	692	T	Cls	0.036	-	-
NR-AhR	NR-AhR	6678	5342	668	668	T	Cls	0.115	-	-
NR-Aromatase	NR-Aromatase	5950	4759	596	595	T	Cls	0.044	-	-
NR-ER	NR-ER	6220	4976	622	622	T	Cls	0.049	-	-
NR-ER-LBD	NR-ER-LBD	7124	5698	713	713	T	Cls	0.108	-	-
NR-PPAR-gamma	NR-PPAR-gamma	6647	5317	665	665	T	Cls	0.030	-	-
PPB	PPB	4793	3834	480	479	D	Reg	-	0.789	0.276
Pgp-inhibitor	Pgp-inh	2255	1804	226	225	A	Cls	0.599	-	-
Pgp-substrate	Pgp-sub	1248	998	125	125	A	Cls	0.514	-	-
Rat Oral Acute Toxicity	ROA	7342	5873	735	734	T	Cls	0.382	-	-
Respiratory Toxicity	Respiratory	1397	1117	140	140	T	Cls	0.603	-	-
SR-ARE	SR-ARE	5667	4533	567	567	T	Cls	0.153	-	-
SR-ATAD5	SR-ATAD5	7236	5789	723	724	T	Cls	0.035	-	-
SR-HSE	SR-HSE	6336	5068	634	634	T	Cls	0.057	-	-
SR-MMP	SR-MMP	5984	4786	599	599	T	Cls	0.152	-	-
SR-p53	SR-p53	7031	5624	704	703	T	Cls	0.067	-	-
Skin Sensitization	SkinSen	405	324	41	40	T	Cls	0.676	-	-
$T_{1/2}$	$T_{1/2}$	1280	1136	83	61	E	Cls	0.406	-	-
VD	VD	1118	895	111	112	D	Reg	-	1.20	1.52
hERG	hERG	13845	11076	1385	1384	T	Cls	0.500	-	-
R/S Chirality	R/S	75048	50032	0	12508	C	Cls	0.500	-	-
Rotatory Strength	Rotatory Strength	131572	105257	13157	13158	C	Reg	-	0.00049	0.06330
Chiral Cliff	Chiral Cliff	3828	3062	0	766	C	Cls	0.470	-	-

9 Training Configurations

To facilitate the replication in the Results section, we outline the critical training configurations and hyperparameters in Supplementary Table 8. In our ADMET-P prediction experiments, we consistently utilize 500,000 update steps for mixed pretraining across various tasks, which spanned around 120 hours on a 4x NVIDIA A100 GPU setup. When fine-tuning the model for specific tasks or training a separate model for each task from scratch, the total number of update steps is set to 20,000. For chirality prediction tasks, as detailed in the right part of Supplementary Table 8, we implement slight modifications in the batch size, warmup updates, and the maximum number of update steps to optimize performance.

Supplementary Table 8 Model Configurations

**Hyperparameter**	**A**	**B**	**C**	**D**	**E**	**F**	R/S Chirality	Chiral Cliff	Chiral Cliff (FT)	Rotatory Strength
Mixed training	×	√	√	×	√	×	×	×	×	×
SE(3)-encoder	√	×	√	√	√	√	√	√	√	√
Meta encoder	√	×	×	×	√	√	√	√	√	√
Fine-tune	×	×	×	√	×	√	×	×	√	×
Optimizer	adam						adam			
Learning rate	$1e-5$						$1e-5$			
Weight decay	0.001						0.001			
Embedding dimension	768						768			
FFN embedding dimension	768						768			
Attention heads	48						48			
Layers	12						12			
Blocks	4						4			
GBF kernels	128						128			
Node loss weight	15						15			
Edge loss weight	25						25			
Geometry perturbation STD. (Å)	0.2						0.2			
Geometry update freq	6						6			
Number of experts	8						8			
Batch size	32						64	8	8	64
Warmup updates	500	5000	5000	500	5000	500	5000	5000	5000	1645
Max update	20000	500000	500000	20000	500000	20000	32000	500000	500000	164600
Classification weight	-	5	5	-	5	-	5	1	1	-
Dataset balancing factor	-	0.2	0.2	-	0.2	-	-	-	-	-
Load checkpoint	-	-	-	**C**	-	**E**	-	-	**E**	-

10 Similarity Info of Dataset

To conduct a more detailed analysis of the entire dataset, we calculated the molecular similarity for each endpoint. The similarity is defined as the average distance of all molecules in training set to their nearest molecules in the testing/validation set, respectively. As shown in Supplementary Table 9, specifically, we computed the Tanimoto similarity between the training and testing sets, as well as between the training and validation sets for each endpoint. The similarity calculations included three methods: based on Morgan fingerprints with the radius set to 2, which were finally folded into either 2048 bits or 128 bits, the last method is based on MACCS fingerprints. By analyzing all the similarity results, we have drawn the following conclusions regarding the dataset and set groups: (i) The similarity differences between different endpoints are significant, This is mainly attributed to the significant structural variations of the molecules related to different tasks. (ii) Within the same endpoint, the similarity differences between the training/testing and training/validation sets are very small. This further indicates that we used a completely random 8:1:1 grouping. (iii) The similarity calculated using MACCS fingerprints is significantly higher than that calculated using Morgan fingerprints. This is mainly because MACCS fingerprints are primarily based on functional group properties, while Morgan fingerprints are based on topological properties. This indicates that the molecules within the same endpoint have strong associations with functional groups, which further highlights the importance of attention distribution. (iv) The similarity of the 2048 bits Morgan fingerprints is noticeably

lower than that of the 128 bits fingerprints. This may be because the 2048 bits Morgan fingerprints can capture more molecular features, but this can also introduce noise during comparisons.

Supplementary Table 9 Similarity Info

Endpoint category	Endpoint	Similarity (ECFP, 2048 bits)		Similarity (ECFP, 128 bits)		Similarity (MACCS)	
		Train & test	Train & valid	Train & test	Train & valid	Train & test	Train & valid
Absorption (A)	Pgp-inh	0.4510	0.4550	0.5614	0.5650	0.7708	0.7693
	Pgp-sub	0.3088	0.2943	0.4609	0.4538	0.6747	0.6703
	HIA	0.3040	0.3059	0.4381	0.4377	0.6560	0.6530
	F20%	0.2939	0.2984	0.4330	0.4391	0.6593	0.6662
	F30%	0.2906	0.2919	0.4309	0.4352	0.6526	0.6585
	Caco-2	0.3684	0.4475	0.4954	0.5578	0.7020	0.7525
	MDCK	0.4065	0.4507	0.5479	0.5787	0.7581	0.7756
Distribution (D)	BBBP	0.3831	0.3718	0.5013	0.4939	0.7220	0.7178
	PPB	0.4035	0.4066	0.5394	0.5399	0.7500	0.7534
	VD	0.3336	0.3474	0.4390	0.4874	0.6965	0.7026
	Fu	0.4066	0.4107	0.5517	0.5528	0.7552	0.7616
Metabolism (M)	CYP1A2-inh	0.4252	0.4272	0.5419	0.5413	0.7649	0.7643
	CYP1A2-sub	0.2263	0.2168	0.3735	0.3651	0.5711	0.5587
	CYP2C19-inh	0.4293	0.4309	0.5442	0.5456	0.7669	0.7673
	CYP2C19-sub	0.2248	0.2061	0.3689	0.3546	0.5590	0.5428
	CYP2C9-inh	0.4283	0.4291	0.5439	0.5447	0.7633	0.7630
	CYP2C9-sub	0.2873	0.2972	0.4279	0.4340	0.6415	0.6472
	CYP2D6-inh	0.4293	0.4265	0.5440	0.5413	0.7657	0.7665
	CYP2D6-sub	0.3039	0.2899	0.4390	0.4277	0.6542	0.6267
	CYP3A4-inh	0.4218	0.4215	0.5378	0.5384	0.7615	0.7625
	CYP3A4-sub	0.2648	0.2673	0.4168	0.4237	0.6363	0.6457
Excretion (E)	T1/2	0.2562	0.2714	0.4274	0.4390	0.6364	0.6583
Toxicity	hERG inhibition	0.3759	0.3728	0.5193	0.5162	0.7556	0.7539
	H-HT	0.3499	0.3520	0.4760	0.4810	0.6958	0.6977
	DILI	0.2490	0.2329	0.3882	0.3767	0.5947	0.5928
	Ames	0.4525	0.4577	0.5246	0.5276	0.7671	0.7677
	ROA	0.4116	0.4152	0.4933	0.4948	0.7172	0.7158
	FDAMDD	0.3288	0.3215	0.4487	0.4436	0.6585	0.6664
	SkinSen	0.3265	0.3107	0.4196	0.4196	0.5814	0.5682
	Carcinogenicity	0.3035	0.3168	0.3955	0.4085	0.6121	0.6198
	EC	0.3585	0.3617	0.4549	0.4588	0.6787	0.6825
	EI	0.4175	0.4092	0.4953	0.4867	0.7267	0.7201
	Respiratory	0.3698	0.3637	0.4761	0.4720	0.6807	0.6769
	NR-AR	0.4089	0.4127	0.4957	0.4982	0.7196	0.7229
	NR-AR-LBD	0.4126	0.4115	0.4974	0.4973	0.7216	0.7217
	NR-AhR	0.4107	0.4077	0.4958	0.4946	0.7234	0.7186
	NR-Aromatase	0.4012	0.4101	0.4879	0.4960	0.7128	0.7156
	NR-ER	0.4043	0.4123	0.4890	0.4963	0.7154	0.7110
	NR-ER-LBD	0.4152	0.4154	0.5009	0.5021	0.7223	0.7227
	NR-PPAR-\gamma	0.4119	0.4152	0.4963	0.5002	0.7208	0.7218
	SR-ARE	0.4074	0.4067	0.4904	0.4883	0.7111	0.7133
	SR-ATAD5	0.4132	0.4115	0.4984	0.4981	0.7217	0.7219
	SR-HSE	0.4095	0.4044	0.4934	0.4883	0.7134	0.7153
	SR-MMP	0.4107	0.4000	0.4959	0.4854	0.7173	0.7104
	SR-p53	0.4150	0.4161	0.4993	0.5012	0.7212	0.7237
	BCF	0.3323	0.3334	0.3979	0.4022	0.6529	0.6508
	IGC$_{50}$	0.4609	0.4616	0.5123	0.5162	0.7331	0.7409
	LC$_{50}FM$	0.3532	0.3432	0.4198	0.4030	0.6181	0.6160
	LC$_{50}DM$	0.3163	0.3142	0.3778	0.3775	0.5843	0.5661
Physiochemical property (P)	LogS	0.4247	0.4351	0.5672	0.5701	0.7335	0.7343
	LogD	0.5069	0.5095	0.6141	0.6165	0.8107	0.8107
	LogP	0.4973	0.5024	0.4944	0.5034	0.7911	0.7919

11 Physiochemical Descriptors of Dataset

In order to gain a clearer understanding of the entire dataset, we calculated its key physicochemical descriptors. Specifically, as shown in Supplementary Table 10, we computed seven physicochemical descriptors for the training, testing, and validation sets within each endpoint, respectively. The seven physicochemical descriptors include Molecular weight (MW), LogP, Hydrogen Bond Donors (HBD), Hydrogen Bond Acceptors (HBA), Topological Polar Surface Area (TPSA), Rotatable Bonds (RB), Number of Rings (NR), which are vital for virtual screening. For each value in the result, the number outside the parentheses represents the mean, while the number inside the parentheses represents the standard deviation. Through Supplementary Table 10, we can clearly see that for each physicochemical descriptor, the training, testing, and validation sets within the same endpoint are very similar; however, there are significant differences between different endpoints.

Supplementary Table 10 Physiochemical Descriptors

Endpoint	MW			LogP			HBD			HBA			TPSA			RB			NR		
	training	test	val	training	test	val	training	test	val	training	test	val	training	test	val	training	test	val	training	test	val
Ames	241 (106)	251 (115)	248 (109)	2.4 (2.0)	2.6 (2.0)	2.6 (2.1)	1.1 (1.3)	1.1 (1.4)	1.1 (1.4)	3.3 (2.3)	3.5 (2.4)	3.3 (2.5)	57 (42)	59 (44)	56 (43)	2.8 (3.2)	2.9 (3.5)	2.9 (3.4)	2.0 (1.6)	2.1 (1.6)	2.2 (1.6)
BBB	360 (160)	353 (161)	361 (186)	2.4 (2.2)	2.2 (2.2)	2.4 (2.3)	1.8 (2.1)	1.8 (2.0)	1.8 (2.2)	4.9 (3.4)	4.9 (3.4)	4.9 (3.4)	79 (65)	79 (62)	80 (67)	4.7 (3.8)	4.5 (3.3)	4.9 (4.5)	3.0 (1.6)	2.9 (1.6)	3.0 (1.6)
BCF	233 (113)	253 (106)	227 (101)	3.4 (2.0)	3.2 (2.2)	3.3 (2.1)	0.6 (0.9)	0.7 (1.0)	0.9 (0.9)	1.8 (2.0)	2.6 (2.3)	2.0 (1.7)	31 (34)	43 (41)	37 (35)	2.5 (3.7)	3.2 (4.6)	3.2 (4.9)	1.4 (1.1)	1.4 (1.0)	1.4 (1.1)
Caco-2	405 (153)	349 (138)	353 (141)	2.2 (2.0)	1.6 (2.0)	1.9 (1.8)	2.4 (2.0)	2.4 (1.6)	2.0 (1.5)	5.7 (3.0)	4.7 (2.2)	4.9 (2.4)	99 (53)	93 (48)	90 (45)	5.5 (3.8)	4.4 (3.4)	4.8 (3.3)	3.2 (1.6)	2.5 (1.5)	2.5 (1.5)
Carcinogenicity	243 (137)	258 (170)	252 (132)	2.0 (2.2)	2.3 (2.4)	2.0 (2.2)	1.1 (1.3)	1.1 (1.2)	1.1 (1.4)	3.6 (2.8)	3.6 (3.0)	3.9 (2.7)	61 (49)	63 (47)	66 (44)	3.3 (3.4)	4.1 (5.3)	3.4 (3.4)	1.7 (1.4)	1.6 (1.3)	1.7 (1.7)
CYP1A2-inh	351 (105)	353 (113)	347 (105)	2.9 (1.9)	2.8 (2.0)	2.9 (1.8)	1.2 (1.2)	1.3 (1.4)	1.2 (1.2)	4.9 (2.2)	4.9 (2.4)	4.8 (2.2)	73 (37)	74 (41)	72 (37)	4.5 (2.6)	4.5 (2.4)	4.4 (2.6)	3.1 (1.3)	3.1 (1.3)	3.1 (1.4)
CYP1A2-sub	336 (181)	341 (145)	388 (183)	2.4 (2.7)	2.1 (2.1)	2.8 (1.9)	2.0 (2.0)	1.8 (1.4)	1.9 (2.0)	4.6 (3.4)	4.9 (3.1)	4.7 (2.6)	80 (67)	81 (52)	77 (53)	4.9 (5.9)	5.5 (3.5)	5.5 (4.1)	2.5 (1.5)	2.6 (1.5)	3.4 (2.0)
CYP2C19-inh	351 (103)	349 (108)	348 (98)	3.0 (1.8)	3.0 (1.8)	3.0 (1.8)	1.2 (1.2)	1.2 (1.3)	1.2 (1.1)	4.8 (2.2)	4.7 (2.2)	4.8 (2.2)	72 (35)	70 (38)	71 (34)	4.5 (2.5)	4.5 (2.5)	4.5 (2.6)	3.1 (1.3)	3.2 (1.4)	3.1 (1.3)
CYP2C19-sub	327 (151)	348 (152)	362 (317)	2.3 (2.5)	1.7 (2.4)	1.8 (2.3)	1.8 (1.8)	2.3 (1.9)	2.9 (4.2)	4.4 (3.5)	5.4 (3.9)	5.3 (5.9)	77 (62)	96 (70)	100 (124)	4.7 (4.2)	5.0 (3.5)	4.1 (4.5)	2.5 (1.6)	2.6 (1.5)	3.0 (2.8)
CYP2C9-inh	353 (109)	348 (105)	355 (115)	2.8 (2.0)	2.8 (2.0)	2.8 (1.9)	1.3 (1.3)	1.2 (1.4)	1.2 (1.2)	4.9 (2.3)	4.8 (2.4)	4.8 (2.2)	74 (39)	73 (40)	73 (38)	4.5 (2.6)	4.5 (2.6)	4.5 (2.5)	3.1 (1.4)	3.1 (1.3)	3.1 (1.3)
CYP2C9-sub	338 (141)	324 (108)	351 (138)	3.0 (1.6)	3.2 (1.7)	3.2 (1.5)	1.4 (1.3)	1.2 (1.2)	1.2 (1.2)	4.2 (2.8)	3.8 (2.2)	4.3 (2.8)	65 (44)	57 (33)	64 (44)	4.4 (3.1)	4.7 (3.2)	4.6 (3.7)	2.9 (1.5)	2.8 (1.4)	3.2 (1.8)
CYP2D6-inh	348 (103)	350 (102)	356 (122)	2.9 (1.8)	2.9 (1.8)	2.9 (1.9)	1.2 (1.2)	1.2 (1.2)	1.3 (1.5)	4.8 (2.2)	4.9 (2.3)	4.9 (2.5)	72 (35)	73 (37)	75 (42)	4.5 (2.5)	4.5 (2.6)	4.5 (2.7)	3.1 (1.3)	3.1 (1.4)	3.2 (1.5)
CYP2D6-sub	332 (135)	345 (172)	323 (141)	3.0 (1.6)	3.0 (1.6)	2.7 (1.7)	1.3 (1.3)	1.4 (1.3)	1.2 (1.1)	4.1 (2.6)	4.3 (3.3)	4.2 (2.6)	61 (42)	65 (48)	62 (43)	4.3 (3.1)	4.4 (3.1)	5.6 (3.6)	2.9 (1.6)	2.9 (1.8)	2.4 (1.3)
CYP3A4-inh	348 (105)	350 (110)	346 (111)	2.9 (1.8)	2.9 (1.9)	2.9 (1.8)	1.2 (1.2)	1.4 (1.4)	1.2 (1.3)	4.9 (2.2)	4.8 (2.3)	4.8 (2.2)	73 (36)	73 (38)	72 (36)	4.6 (2.6)	4.6 (2.4)	4.6 (2.6)	3.1 (1.4)	3.1 (1.4)	3.0 (1.4)
CYP3A4-sub	380 (187)	374 (181)	468 (639)	2.5 (2.6)	2.8 (2.9)	1.6 (9.4)	2.2 (2.3)	2.1 (2.2)	3.6 (12.0)	5.3 (3.8)	4.7 (3.2)	7.5 (19.6)	91 (70)	85 (73)	130 (322)	5.6 (5.3)	5.3 (5.1)	7.4 (16.3)	3.0 (1.8)	3.1 (1.6)	3.5 (3.8)
DILI	343 (173)	314 (129)	342 (167)	2.1 (2.7)	2.2 (2.5)	2.3 (2.8)	2.2 (2.4)	1.8 (1.5)	1.7 (2.9)	5.0 (3.6)	4.3 (2.8)	4.8 (3.5)	84 (66)	73 (45)	82 (79)	4.8 (4.0)	4.4 (3.4)	5.7 (5.3)	2.6 (1.5)	2.5 (1.5)	2.4 (1.3)
EC	272 (137)	279 (136)	266 (134)	2.2 (2.0)	2.4 (2.0)	2.2 (2.2)	1.4 (1.5)	1.4 (1.5)	1.3 (1.7)	3.4 (2.6)	3.3 (2.7)	3.3 (2.9)	58 (46)	56 (49)	56 (49)	4.0 (3.6)	4.1 (3.5)	3.8 (3.4)	1.9 (1.6)	1.9 (1.6)	1.9 (1.6)
EI	223 (114)	225 (116)	223 (121)	2.1 (1.8)	2.2 (1.7)	2.1 (1.8)	1.1 (1.3)	1.0 (1.2)	1.1 (1.4)	2.7 (2.1)	2.7 (2.1)	2.7 (2.1)	49 (39)	49 (38)	48 (39)	2.9 (3.4)	3.0 (3.6)	2.8 (3.4)	1.5 (1.3)	1.4 (1.3)	1.5 (1.3)
F(20%)	357 (162)	379 (217)	359 (175)	2.1 (2.3)	2.5 (2.9)	2.5 (2.1)	2.1 (2.3)	2.4 (3.1)	2.0 (2.4)	5.1 (3.5)	5.1 (4.4)	4.9 (3.1)	86 (65)	89 (86)	83 (75)	5.0 (3.7)	5.1 (4.4)	5.1 (4.4)	2.8 (1.6)	3.2 (2.1)	2.9 (1.4)
F(30%)	356 (171)	374 (177)	369 (149)	2.2 (2.4)	2.4 (2.2)	2.4 (2.7)	2.2 (2.5)	2.0 (2.0)	2.0 (2.0)	5.1 (3.6)	5.2 (3.1)	5.3 (3.2)	86 (70)	90 (70)	85 (56)	5.0 (3.8)	5.1 (3.9)	5.0 (3.9)	2.9 (1.7)	3.0 (1.6)	2.9 (1.5)
FDAMDD	345 (167)	335 (152)	361 (186)	2.2 (2.5)	1.9 (2.6)	1.9 (2.7)	1.9 (2.0)	1.9 (2.2)	2.3 (2.5)	4.8 (3.4)	4.7 (3.2)	5.7 (4.4)	80 (60)	76 (62)	93 (73)	4.6 (3.5)	4.8 (4.2)	5.3 (4.1)	2.6 (1.6)	2.6 (1.7)	2.7 (1.7)
Fu	438 (160)	439 (146)	430 (131)	3.3 (2.0)	3.3 (1.9)	3.4 (1.9)	2.1 (2.2)	2.2 (2.2)	2.0 (1.8)	5.8 (3.1)	6.0 (3.1)	5.6 (2.4)	97 (63)	100 (64)	93 (46)	5.8 (3.4)	5.7 (2.7)	5.5 (3.4)	3.8 (1.5)	4.0 (1.6)	3.8 (1.4)
H-HT	404 (426)	397 (319)	458 (584)	2.1 (3.1)	2.2 (3.6)	2.7 (3.3)	2.7 (5.5)	2.7 (4.6)	3.4 (9.4)	5.9 (8.5)	5.8 (5.8)	6.1 (9.2)	107 (178)	106 (139)	125 (273)	6.2 (11.4)	6.5 (10.1)	7.3 (16.0)	2.9 (2.3)	2.8 (2.1)	3.1 (1.8)
hERG	379 (94)	378 (91)	377 (93)	3.5 (1.5)	3.4 (1.5)	3.5 (1.5)	1.2 (1.1)	1.2 (1.0)	1.2 (1.0)	4.9 (2.0)	5.0 (1.9)	4.9 (1.9)	68 (30)	68 (30)	66 (30)	5.0 (2.6)	5.1 (2.6)	5.1 (2.6)	3.5 (1.3)	3.5 (1.3)	3.4 (1.3)
HIA	344 (161)	351 (147)	351 (188)	2.1 (2.3)	2.3 (2.4)	2.2 (2.7)	2.0 (2.2)	1.9 (2.3)	2.2 (2.5)	4.8 (3.4)	4.8 (3.4)	4.8 (3.5)	83 (65)	83 (65)	80 (66)	4.7 (3.7)	5.1 (3.6)	5.2 (4.7)	2.7 (1.6)	2.8 (1.5)	2.7 (1.7)
IGC50	151 (50)	153 (53)	153 (46)	1.8 (1.1)	1.7 (1.0)	1.9 (1.0)	0.6 (0.7)	0.6 (0.7)	0.6 (0.6)	1.7 (1.1)	1.9 (1.1)	1.7 (1.0)	32 (21)	35 (22)	32 (21)	2.0 (2.1)	2.0 (1.8)	2.2 (2.2)	0.7 (0.7)	0.7 (0.6)	0.7 (0.6)
LC50	180 (86)	195 (105)	178 (69)	2.2 (1.6)	2.3 (1.7)	2.1 (1.3)	0.6 (0.8)	0.7 (0.7)	0.4 (0.6)	2.1 (1.7)	2.2 (1.9)	2.2 (1.6)	33 (27)	39 (30)	35 (27)	2.5 (2.8)	2.5 (2.9)	2.2 (2.1)	0.9 (0.9)	1.1 (1.1)	0.9 (0.8)
LC50DM	199 (107)	194 (119)	186 (82)	2.5 (1.8)	2.6 (2.2)	2.4 (1.6)	0.6 (0.8)	0.7 (1.0)	0.5 (0.7)	2.1 (2.1)	1.7 (1.8)	1.7 (1.4)	33 (32)	28 (30)	30 (29)	2.1 (3.0)	1.7 (2.9)	1.9 (2.2)	1.2 (1.2)	1.1 (1.1)	1.1 (1.1)
LogD	396 (107)	395 (106)	400 (112)	3.1 (1.4)	3.0 (1.5)	3.1 (1.5)	1.3 (1.1)	1.2 (1.1)	1.3 (1.2)	5.2 (2.2)	5.3 (2.2)	5.3 (2.3)	81 (33)	81 (33)	81 (36)	5.4 (3.2)	5.3 (3.0)	5.5 (3.3)	3.6 (1.2)	3.6 (1.2)	3.6 (1.2)
LogP	247 (108)	241 (100)	249 (107)	2.1(1.6)	2.1(1.5)	2.1(1.7)	1.1 (1.2)	1.0 (1.1)	1.1 (1.2)	3.4 (2.3)	3.3 (2.2)	3.3 (2.3)	57 (38)	55 (36)	56 (37)	3.2 (2.9)	3.1 (2.8)	3.2 (3.0)	1.7 (1.1)	1.7 (1.1)	1.7 (1.2)
LogS	220 (104)	225 (102)	226 (103)	2.1 (2.0)	2.1 (2.1)	2.2 (2.2)	1.0 (1.2)	1.1 (1.3)	1.1 (1.3)	2.9 (2.2)	2.9 (2.2)	2.9 (2.4)	51 (40)	55 (42)	53 (45)	2.9 (3.0)	3.3 (3.3)	3.1 (3.3)	1.3 (1.2)	1.3 (1.2)	1.4 (1.2)
MDCK	395 (65)	401 (60)	402 (65)	3.2 (1.1)	3.2 (1.1)	3.3 (1.1)	1.7 (1.0)	1.8 (1.0)	1.6 (1.0)	5.4 (1.6)	5.4 (1.7)	5.3 (1.6)	83 (27)	82 (22)	83 (26)	4.7 (2.1)	5.3 (2.1)	4.8 (2.1)	3.8 (0.9)	3.8 (1.0)	3.9 (0.9)
NR-AhR	270 (163)	274 (178)	269 (164)	2.3 (2.3)	2.3 (2.3)	2.4 (2.3)	1.2 (1.9)	1.4 (2.0)	1.2 (1.9)	3.5 (3.2)	3.6 (3.3)	3.3 (3.2)	59 (58)	61 (63)	57 (63)	4.3 (4.4)	4.5 (5.5)	4.2 (4.6)	1.7 (1.7)	1.7 (1.6)	1.7 (1.5)
NR-AR-LBD	266 (157)	272 (155)	272 (176)	2.3 (2.2)	2.3 (2.5)	2.3 (2.3)	1.2 (1.9)	1.3 (1.9)	1.3 (2.3)	3.4 (3.0)	3.7 (3.5)	3.6 (3.6)	58 (57)	61 (61)	61 (67)	4.2 (4.3)	4.5 (4.6)	4.5 (4.9)	1.7 (1.6)	1.7 (1.6)	1.7 (1.8)
NR-AR	274 (160)	278 (171)	278 (169)	2.4 (2.3)	2.4 (2.2)	2.5 (2.3)	1.2 (1.9)	1.2 (1.9)	1.2 (1.9)	3.5 (3.1)	3.6 (3.3)	3.5 (3.2)	59 (57)	60 (57)	59 (62)	4.4 (4.5)	4.3 (5.0)	4.3 (4.6)	1.7 (1.6)	1.9 (1.9)	1.8 (1.6)
NR-Aromatase	262 (162)	261 (159)	264 (163)	2.2 (2.3)	2.2 (2.3)	1.9 (2.4)	1.3 (1.9)	1.2 (1.9)	1.4 (2.2)	3.4 (3.1)	3.5 (3.3)	3.8 (3.4)	58 (59)	58 (57)	64 (61)	4.3 (4.6)	4.1 (4.0)	4.2 (4.0)	1.6 (1.5)	1.7 (1.9)	1.6 (1.7)
NR-ER-LBD	271 (161)	272 (169)	263 (152)	2.3 (2.2)	2.4 (2.5)	2.3 (2.2)	1.3 (1.9)	1.3 (2.0)	1.2 (1.8)	3.5 (3.2)	3.6 (3.3)	3.4 (3.2)	59 (58)	58 (61)	57 (54)	4.3 (4.4)	4.4 (4.8)	4.0 (4.0)	1.7 (1.6)	1.7 (1.5)	1.7 (1.8)
NR-ER	262 (156)	268 (159)	262 (159)	2.3 (2.2)	2.3 (2.3)	2.2 (2.3)	1.2 (1.9)	1.3 (1.9)	1.2 (1.8)	3.4 (3.2)	3.6 (3.2)	3.5 (2.7)	58 (59)	60 (56)	59 (54)	4.2 (4.3)	4.3 (4.6)	4.2 (4.4)	1.7 (1.6)	1.8 (1.6)	1.6 (1.4)
NR-PPAR-gamma	263 (157)	250 (150)	256 (146)	2.2 (2.3)	2.1 (2.1)	2.3 (2.1)	1.2 (1.9)	1.2 (1.8)	1.2 (1.8)	3.5 (3.1)	3.4 (3.2)	3.3 (2.7)	58 (58)	56 (58)	57 (53)	4.2 (4.4)	4.0 (4.1)	3.9 (4.0)	1.6 (1.6)	1.6 (1.5)	1.6 (1.5)
Pgp-inh	422 (170)	393 (150)	405 (158)	3.8 (2.1)	3.7 (2.0)	3.6 (2.0)	1.5 (1.8)	1.3 (1.4)	1.5 (1.5)	5.3 (3.1)	4.9 (2.5)	4.9 (2.8)	75 (59)	68 (46)	73 (51)	6.8 (4.0)	6.4 (4.0)	6.6 (4.0)	3.5 (1.5)	3.3 (1.4)	3.2 (1.5)
Pgp-sub	469 (225)	476 (278)	463 (253)	3.7 (2.1)	4.5 (2.9)	3.7 (2.5)	2.1 (2.2)	2.0 (2.9)	2.1 (2.1)	6.4 (4.6)	5.7 (4.5)	6.8 (4.4)	102 (80)	91 (93)	107 (82)	6.1 (5.3)	7.0 (8.0)	6.3 (5.8)	4.0 (2.0)	4.0 (2.0)	3.7 (2.2)
PPB	412 (154)	421 (168)	403 (162)	3.1 (2.1)	3.2 (1.9)	3.0 (2.2)	1.9 (2.0)	2.0 (2.3)	1.9 (2.2)	5.6 (3.1)	5.5 (3.2)	5.4 (3.2)	92 (60)	92 (63)	89 (67)	5.4 (3.5)	5.4 (3.6)	5.1 (3.4)	3.5 (1.6)	3.6 (1.7)	3.5 (1.6)
Respiratory	295 (118)	286 (109)	302 (121)	2.3 (1.6)	2.3 (1.7)	2.5 (1.8)	1.0 (1.5)	1.2 (1.5)	1.1 (1.3)	4.1 (2.4)	3.9 (2.3)	4.2 (2.6)	59 (42)	61 (38)	58 (39)	3.7 (2.8)	3.6 (2.9)	4.0 (3.0)	2.4 (1.5)	2.4 (1.5)	2.4 (1.6)
ROA	247 (114)	252 (120)	248 (116)	2.5 (1.8)	2.5 (1.9)	2.4 (1.8)	0.8 (1.1)	0.8 (1.3)	0.8 (1.1)	3.3 (2.3)	3.4 (2.4)	3.3 (2.2)	48 (35)	50 (39)	49 (36)	4.0 (3.5)	4.0 (3.6)	3.9 (3.4)	1.4 (1.3)	1.4 (1.3)	1.4 (1.3)
SkinSen	224 (116)	261 (166)	228 (99)	2.5 (2.2)	3.0 (2.0)	2.5 (1.8)	0.9 (1.4)	1.0 (1.3)	0.8 (1.0)	2.7 (2.2)	2.6 (2.6)	2.8 (1.8)	45 (39)	45 (40)	49 (33)	4.2 (4.1)	5.3 (4.6)	3.5 (4.3)	1.2 (1.3)	1.4 (1.7)	1.4 (1.2)
SR-ARE	245 (134)	250 (133)	242 (116)	2.3 (2.0)	2.4 (2.0)	2.3 (2.0)	1.1 (1.5)	1.0 (1.2)	1.0 (1.2)	3.2 (2.6)	3.2 (2.3)	3.0 (2.2)	53 (46)	53 (38)	50 (38)	3.9 (3.9)	4.1 (3.9)	3.8 (3.8)	1.5 (1.5)	1.5 (1.3)	1.5 (1.4)
SR-ATAD5	270 (161)	264 (142)	259 (146)	2.3 (2.2)	2.4 (2.5)	2.3 (2.2)	1.3 (1.9)	1.2 (1.8)	1.2 (1.8)	3.5 (3.1)	3.5 (2.8)	3.5 (3.1)	59 (58)	58 (53)	58 (53)	4.3 (4.4)	4.2 (4.4)	4.1 (4.7)	1.7 (1.6)	1.7 (1.4)	1.7 (1.7)
SR-HSE	253 (134)	248 (131)	248 (135)	2.4 (2.0)	2.4 (2.0)	2.2 (2.2)	1.1 (1.4)	1.0 (1.3)	1.1 (1.7)	3.2 (2.5)	3.2 (2.3)	3.3 (3.0)	53 (44)	54 (43)	55 (49)	3.9 (3.9)	4.0 (3.8)	3.8 (3.6)	1.6 (1.5)	1.5 (1.4)	1.6 (1.8)
SR-MMP	264 (161)	260 (158)	268 (177)	2.2 (2.3)	2.2 (2.3)	2.1 (2.2)	1.3 (1.9)	1.2 (1.9)	1.3 (2.1)	3.5 (3.2)	3.4 (3.0)	3.7 (3.6)	59 (58)	59 (58)	63 (67)	4.3 (4.5)	4.2 (4.4)	4.1 (4.5)	1.6 (1.6)	1.6 (1.5)	1.8 (1.8)
SR-p53	266 (157)	271 (156)	279 (195)	2.3 (2.2)	2.4 (2.1)	2.2 (2.5)	1.3 (1.9)	1.2 (1.6)	1.4 (2.5)	3.5 (3.1)	3.5 (3.0)	3.8 (4.0)	59 (58)	59 (52)	65 (77)	4.2 (4.3)	4.3 (4.4)	4.4 (5.0)	1.7 (1.6)	1.8 (1.6)	1.8 (1.9)
T12	436 (299)	387 (198)	440 (282)	2.0 (2.7)	2.0 (2.3)	1.5 (2.8)	3.0 (4.4)	2.4 (2.5)	3.3 (4.3)	6.4 (5.1)	5.4 (3.6)	6.9 (5.4)	115 (122)	94 (76)	126 (122)	6.6 (7.8)	5.5 (4.5)	6.9 (8.0)	3.3 (2.2)	3.0 (1.5)	3.1 (1.6)
VDss	401 (231)	396 (260)	432 (393)	1.5 (2.5)	1.5 (2.7)	1.3 (3.3)	2.9 (3.4)	3.0 (3.8)	3.5 (6.5)	6.2 (4.3)	6.0 (4.6)	6.7 (6.1)	112 (93)	111 (112)	129 (180)	5.8 (5.9)	6.0 (5.9)	6.8 (12.7)	3.2 (2.1)	3.0 (1.5)	2.9 (1.6)

12 Additional Evaluation with Alignment Operations

To explore the full potential of our model's performance, we conducted additional experiments incorporating alignment operations for minimizing MAE and RMSE metrics. Supplementary Table 11 shows the result comparing regression performance with or without alignment operation. While our main results maintain strict evaluation protocols without alignment to ensure fair comparisons, these supplementary results demonstrate the model's capabilities under optimal alignment conditions.

Supplementary Table 11 Alignment for best MAE and RMSE

Task type	Task Name	MAE		RMSE	
		Without Alignment	With Alignment	Without Alignment	With Alignment
Physiochemical property	LogS	0.509	**0.506**	0.741	**0.730**
	LogD	0.290	**0.289**	0.371	**0.370**
	LogP	0.223	**0.223**	0.309	**0.308**
Absorption	Caco-2	0.195	**0.193**	0.275	**0.273**
	MDCK	0.179	**0.179**	0.251	**0.249**
Distribution	ppb	0.058	**0.058**	0.094	**0.093**
	VDss	0.347	**0.343**	0.660	**0.610**
	Fu	0.197	**0.196**	0.310	**0.310**
Toxicity	BCF	0.416	**0.413**	0.591	**0.587**
	IGC50	0.232	**0.230**	0.360	**0.358**
	LC50	0.532	**0.529**	0.782	**0.776**
	LC50DM	0.566	**0.563**	0.821	**0.810**
Mean		0.312	**0.310**	0.464	**0.456**

It is worth noting that while alignment operations can potentially improve performance metrics, our main results do not incorporate these adjustments to maintain evaluation rigor and prevent potential data leakage through alignment processes. The comparison in Supplementary Table 11 shows that alignment operations yield modest improvements in both MAE and RMSE across various ADMET prediction tasks.

13 Benchmarking Against Traditional ADMET Prediction Methods

To provide comprehensive performance benchmarking, we additionally compared OmniMol against both traditional machine learning approaches and state-of-the-art deep learning frameworks, as illustrated in Supplementary Table 12 and Supplementary Table 13. We implemented the widely-used baseline of Random Forest with Morgan fingerprints (radius=2, 2048 bits), which serves as a robust traditional machine learning benchmark.

As shown in Supplementary Table 12 and Supplementary Table 13, OmniMol consistently outperforms both baseline methods across all metrics. For classification tasks, OmniMol achieves a mean ROC-AUC of 0.905, substantially surpassing both ADMETlab 2.0 (MGA framework) (0.863) and Random Forest (0.841). The advantage of OmniMol is even more evident in regression tasks, where it achieves a mean R^2 of 0.844, representing improvements over both ADMETlab 2.0 (0.770) and Random Forest (0.621). This superior performance is consistent across various physicochemical properties and ADMET endpoints.

The traditional Random Forest approach, while providing a reasonable baseline, consistently underperforms compared to both deep learning methods. This performance gap is particularly evident in regression tasks, where Random Forest's mean R^2 (0.621) is substantially lower than both modern approaches, highlighting the advantages of deep learning architectures in capturing complex structure-property relationships.

Supplementary Table 12 Classification performance comparison between OmniMol, ADMETlab 2.0 (MGA), and Random Forest with Morgan fingerprints across various ADMET endpoints.

Task Name	ROC-AUC			ACC			MCC		
	RF	ADMETlab 2.0	OmniMol	RF	ADMETlab 2.0	OmniMol	RF	ADMETlab 2.0	OmniMol
Pgp-inh	**0.943**	0.922	0.942	0.849	0.867	**0.907**	0.685	0.723	**0.799**
Pgp-sub	0.846	0.840	**0.907**	0.776	0.768	**0.840**	0.552	0.538	**0.631**
HIA	0.883	0.866	**0.940**	0.907	0.924	**0.949**	0.491	0.687	**0.697**
F(20%)	0.723	0.833	**0.933**	0.790	0.750	**0.880**	0.366	0.414	**0.617**
F(30%)	0.764	0.848	**0.910**	0.703	0.802	**0.891**	0.308	0.580	**0.678**
BBBP	0.905	0.908	**0.922**	**0.878**	0.862	0.853	0.620	0.718	**0.748**
CYP1A2-inh	0.907	0.928	**0.934**	0.831	0.852	**0.885**	0.660	0.704	**0.791**
CYP1A2-sub	0.822	0.737	**0.976**	0.784	0.649	**0.892**	0.569	0.298	**0.618**
CYP2C19-inh	0.889	0.913	**0.924**	0.814	0.839	**0.857**	0.627	0.679	**0.773**
CYP2C19-sub	0.652	0.758	**0.958**	0.654	0.654	**0.923**	0.272	0.300	**0.517**
CYP2C9-inh	0.888	**0.919**	**0.919**	0.807	0.841	**0.872**	0.546	0.671	**0.735**
CYP2C9-sub	0.763	0.725	**0.902**	0.707	0.707	**0.866**	0.378	0.386	**0.639**
CYP2D6-inh	0.867	0.892	**0.917**	0.866	0.824	**0.898**	0.510	0.558	**0.693**
CYP2D6-sub	0.758	0.847	**0.903**	0.663	0.775	**0.865**	0.326	0.553	**0.743**
CYP3A4-inh	0.885	0.921	**0.922**	0.793	0.832	**0.848**	0.568	0.659	**0.721**
CYP3A4-sub	0.778	0.776	**0.810**	0.733	0.713	**0.772**	0.467	0.437	**0.478**
$T_{1/2}$	0.784	0.801	**0.851**	0.738	0.727	**0.771**	0.435	0.478	**0.525**
hERG	0.937	**0.943**	0.942	0.870	**0.889**	0.887	0.740	0.778	**0.830**
Hepatotoxicity	0.796	**0.814**	0.794	0.707	0.720	**0.728**	0.395	**0.461**	0.415
DILI	0.912	0.924	**0.932**	0.851	0.894	**0.915**	0.702	0.793	**0.807**
Ames	0.894	0.902	**0.907**	0.811	0.807	**0.845**	0.615	0.606	**0.707**
ROA	0.832	**0.853**	0.835	0.755	**0.778**	0.777	0.467	**0.549**	0.547
FDAMDD	0.834	0.804	**0.837**	0.760	0.736	**0.793**	0.519	0.471	**0.522**
SkinSen	0.821	0.707	**0.842**	0.750	0.775	**0.875**	0.393	**0.462**	0.340
Carcinogenicity	0.747	0.788	**0.806**	0.683	0.731	**0.769**	0.365	0.476	**0.507**
EC	0.992	0.983	**0.996**	0.943	0.957	**0.978**	0.883	0.908	**0.980**
EI	0.974	**0.982**	**0.982**	0.931	0.952	**0.962**	0.815	0.876	**0.955**
Respiratory	0.820	0.828	**0.876**	0.757	0.764	**0.821**	0.485	0.514	**0.639**
NR-AR	0.886	0.886	**0.931**	**0.985**	0.890	**0.985**	**0.755**	0.348	0.717
NR-AR-LBD	0.847	0.915	**0.934**	0.978	0.936	**0.983**	0.603	0.472	**0.720**
NR-AhR	0.919	0.943	**0.952**	0.907	0.862	**0.937**	0.426	0.573	**0.731**
NR-Aromatase	0.740	0.852	**0.884**	0.958	0.849	**0.961**	0.251	0.264	**0.320**
NR-ER	0.783	0.771	**0.837**	0.891	0.815	**0.921**	0.210	0.320	**0.498**
NR-ER-LBD	0.827	0.850	**0.915**	0.964	0.903	**0.966**	0.482	0.364	**0.590**
NR-PPAR-Y	0.821	0.893	**0.896**	0.968	0.896	**0.979**	-0.007	0.344	**0.590**
SR-ARE	0.826	0.863	**0.888**	0.864	0.827	**0.901**	0.332	0.469	**0.606**
SR-ATAD5	0.819	**0.874**	0.867	0.971	0.919	**0.975**	**0.394**	0.361	0.365
SR-HSE	0.830	0.907	**0.912**	0.945	0.868	**0.959**	0.182	0.393	**0.598**
SR-MMP	0.883	0.927	**0.953**	0.890	0.897	**0.940**	0.492	0.660	**0.800**
SR-p53	0.835	0.881	**0.911**	0.936	0.841	**0.960**	0.240	0.365	**0.599**
Mean	0.841	0.863	**0.905**	0.834	0.822	**0.890**	0.478	0.530	**0.645**

Supplementary Table 13 Regression performance comparison between OmniMol, ADMETlab 2.0 (MGA), and Random Forest with Morgan fingerprints for physicochemical property prediction.

Task Name	R^2			MAE			RMSE		
	RF	ADMETlab 2.0	OmniMol	RF	ADMETlab 2.0	OmniMol	RF	ADMETlab 2.0	OmniMol
LogS	0.715	0.854	**0.878**	0.866	0.588	**0.509**	1.186	0.850	**0.741**
LogD	0.716	0.892	**0.924**	0.564	0.347	**0.290**	0.751	0.462	**0.371**
LogP	0.789	0.957	**0.964**	0.578	0.256	**0.223**	0.787	0.357	**0.309**
Caco-2	0.543	0.746	**0.886**	0.298	0.222	**0.195**	0.543	0.307	**0.275**
MDCK	0.633	0.731	**0.801**	0.236	0.199	**0.179**	0.339	0.291	**0.251**
ppb	0.582	0.733	**0.856**	0.117	0.083	**0.058**	0.169	0.135	**0.094**
VDss	0.659	0.782	**0.809**	0.560	0.457	**0.347**	0.838	0.670	**0.660**
Fu	0.505	0.763	**0.848**	0.400	0.263	**0.197**	0.530	0.367	**0.310**
BCF	0.586	0.786	**0.800**	0.648	0.435	**0.416**	0.837	0.603	**0.591**
IGC50	0.550	0.723	**0.858**	0.477	0.335	**0.232**	0.632	0.496	**0.360**
LC50	0.604	0.745	**0.789**	0.838	0.643	**0.532**	1.076	0.863	**0.782**
LC50DM	0.572	0.524	**0.716**	0.608	0.692	**0.566**	0.837	0.994	**0.821**
Mean	0.621	0.770	**0.844**	0.516	0.377	**0.312**	0.710	0.533	**0.464**

Additional comparisons with other top-performing methods, including HelixADMET's LiteGEM framework, are provided in Supplementary Table 1. While dataset differences prevent direct inclusion in the main results, these additional comparisons further support the robust performance of our approach across diverse ADMET prediction tasks.

Supplementary Table 14 Performance comparison between OmniMol and the best traditional baseline (AP+BCD:GB) on chiral cliff dataset across multiple metrics.

Metric	Model	Mean	STD
Acc	OmniMol	0.8015	0.0041
	AP+BCD:GB	0.7694	0.0028
MCC	OmniMol	0.5974	0.0083
	AP+BCD:GB	0.5316	0.0059
F1	OmniMol	0.7747	0.0046
	AP+BCD:GB	0.7356	0.0045
McNemar Statistic		3.2550	1.0883
McNemar P value		0.0857	0.0526
Different Predictions Percentage		20.6418	0.9516

14 Statistical Performance Comparison with Traditional Methods

In this section, we conducted the significance test between OmniMol and the best traditional baseline on the Chiral Cliff dataset. We ran both method 6 times. For each time, we calculate the Acc, MCC, F1-score for each method. We also calculate the MyNemar Statistic and McNemar P Value for the comparison of two different methods.

From the result, we observe that OmniMol achieved superior performance with an accuracy of 0.802 ± 0.004 compared to the baseline's 0.769 ± 0.003. Notably, the Matthews Correlation Coefficient (MCC), which is particularly valuable for imbalanced classification tasks, showed a more substantial difference: OmniMol attained an MCC of 0.597 ± 0.008 versus the baseline's 0.531 ± 0.006. The F1 score further confirmed this trend with OmniMol achieving 0.775 ± 0.005 compared to the baseline's 0.736 ± 0.004. The McNemar's test, which assesses the statistical significance of the differences between paired nominal data, yielded a mean p-value of 0.086 ± 0.053. While this value approaches but does not reach the conventional significance threshold of 0.05, it suggests a notable trend in the performance difference between the models. The two models produced divergent predictions for approximately $20.6\% \pm 1.0\%$ of the test cases, indicating substantial complementarity in their decision-making processes.

Importantly, based on the paper of [26], the AP+BCD:GB (gradient boosting on AtomPairs Fingerprints + Best Chiral Descriptors, traditional method) is the best method selected from all other traditional methods with different combinations of learning algorithms (Random forests, logistic regressions) and descriptors and fingerprints. On the contrary, our method is a geometric deep learning framework that requires only SMILES input and MMFF-based conformer generation, eliminating the need for expert-crafted descriptors and complex feature engineering our model automatically learns the essential 3D geometric and chemical features directly from molecular conformations, demonstrating the power of end-to-end learning in capturing chirality-specific characteristics. In conclusion, OmniMol performed statistically on par with the best chiral descriptor based method, without complex designing and selection of descriptors. This indicates the potential of geometric deep learning approaches to surpass traditional methods that rely heavily on hand-crafted descriptors, while offering a more generalizable solution for chiral molecular property prediction.

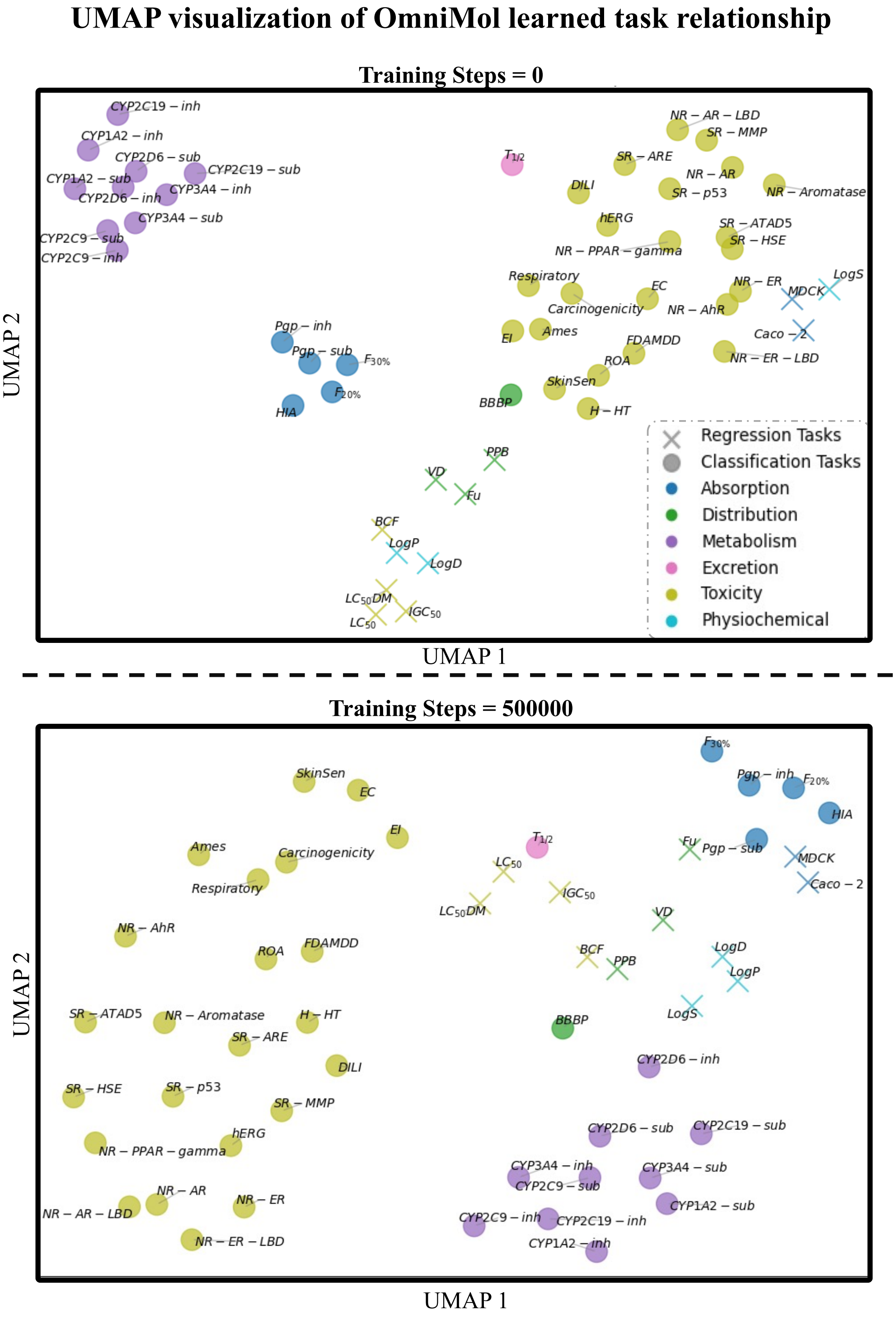

Supplementary Fig. 2 Comparison of initial (top) and learned (bottom) meta-embeddings. The visualization demonstrates the evolution from statistics-based task embeddings to learned representations that capture both ADMET-P categories and prediction types (regression/classification), while revealing biologically meaningful task relationships. Tasks are colored by their ADMET-P categories, with different shapes indicating regression versus classification tasks.

15 Analysis of Meta-Embedding Evolution through Training

We conducted a comparative analysis between the initial and learned meta-embeddings, as illustrated in Supplementary Fig. 2. The upper panel displays the original meta-embeddings derived from dataset statistics, while the lower panel shows the final learned meta-embeddings after model training. This comparison reveals several significant transformations that demonstrate the model's capacity to learn meaningful task relationships:

1. Initially, regression and classification tasks within the same ADMET-P category were disparately positioned in the embedding space. For instance, regression-based toxicity tasks (BCF, LC_{50}) were distinctly separated from classification-based toxicity endpoints (DILI, hERG). Similarly, physicochemical property predictors (LogP, LogD, LogS) and absorption-related tasks were scattered across the space. In contrast, the learned embeddings exhibit clear organizational principles: tasks are clustered primarily by their ADMET-P category while maintaining a natural separation between regression and classification tasks. This organization suggests that the model has learned to recognize both the mechanistic similarities within ADMET-P categories and the fundamental differences in prediction types.
2. The learned embeddings reveal biologically meaningful relationships that were not encoded in the original feature space. A notable example is the positioning of DILI and H-HT tasks, which are known to be mechanistically related due to their involvement in drug-induced liver injury and hepatotoxicity. While these tasks were initially embedded far apart, the learned representation positioned them in close proximity, despite no explicit encoding of this biological relationship in the input features. This demonstrates the model's ability to capture intrinsic task relationships through the learning process.
3. The spatial distribution characteristics of the embeddings underwent significant refinement. The initial embedding space showed heterogeneous clustering, particularly evident in the widely dispersed metabolism-related tasks from majority. The learned embeddings display a more uniform distribution while maintaining clear category boundaries, suggesting an optimization of the embedding space that balances task relationships with effective space utilization.

These transformations from the initial to the final embedding space demonstrate that our model learns meaningful task representations beyond the original dataset statistics. The learned embeddings capture both explicit categorical relationships (ADMET-P groupings) and implicit biological connections (e.g., DILI and H-HT correlation), while maintaining a structured organization that reflects the fundamental nature of the prediction tasks (regression vs. classification). This provides strong evidence that the meta-learning process effectively discovers and encodes relevant task relationships, enhancing the model's ability to transfer knowledge across related tasks.

16 Statistical Validation through Y-Randomization Tests

To validate that our model's performance was not obtained by chance, we conducted Y-randomization tests on 8 datasets that included in our explainability result, for evaluating the statistical significance of our method. In this test, we randomly permuted the targeted properties (Y) while keeping the molecular input unchanged, which effectively destroys any true structure-activity relationships while maintaining the original representation distribution. This experiment directly tests whether the model has learned meaningful structure-property relationships or merely captured random correlations.

The Y-randomization results shown in Supplementary Table 15 provide strong evidence that our model's performance is not due to chance. For classification tasks, the

Supplementary Table 15 Performance comparison between original and Y-randomized data across eight ADMET tasks.

Task Name	ROC-AUC		ACC	
	Original	Y-randomized	Original	Y-randomized
F(20%)	0.933	0.448	0.880	0.740
SkinSen	0.842	0.658	0.875	0.675
Carcinogenicity	0.806	0.537	0.769	0.538
hERG	0.942	0.519	0.887	0.500
Ames	0.907	0.535	0.845	0.562
H-HT	0.794	0.524	0.728	0.573
DILI	0.932	0.563	0.915	0.574
	R^2		MAE	
	Original	Y-randomized	Original	Y-randomized
LogP	0.964	0.004	0.223	1.322

ROC-AUC scores on Y-randomized data consistently fell to near-random levels (0.448-0.658), showing marked deterioration from the original performance (0.794-0.942). This substantial drop in performance is particularly evident in tasks like F(20%) (ROC-AUC: 0.933 → 0.448) and hERG (ROC-AUC: 0.942 → 0.519). The accuracy metrics show similar degradation, with differences ranging from 15.5 to 38.7 percentage points compared to the original models.

The regression task (LogP prediction) demonstrates even more dramatic evidence against chance correlation, with the R^2 value dropping from 0.964 to nearly zero (0.004) under Y-randomization, while the MAE increased almost six-fold from 0.223 to 1.322. This severe performance degradation under Y-randomization is characteristic of models that have successfully captured true structure-property relationships rather than chance correlations.

These results consistently demonstrate that across all tasks, the model's performance collapses to near-random levels when the structure-property relationships are destroyed through Y-randomization, while maintaining strong predictive power on the original data. This pattern provides robust statistical evidence that our model has learned meaningful chemical structure-property relationships rather than achieving its performance by chance.

17 Data Aligned Comparison with HelixADMET Using Scaffold Split

To provide a more comprehensive comparison between our method and HelixADMET, we conducted additional experiments using scaffold splitting, as suggested in the literature [38]. Scaffold splitting aims to evaluate the models on structurally novel compounds by ensuring that compounds in the test set have different scaffolds from those in the training set. This approach provides a more challenging and realistic assessment of a model's generalization ability.

We followed the scaffold splitting procedure described in the HelixADMET paper. Specifically, we used the Bemis-Murcko method implemented in RDKit [19] to extract the scaffolds of compounds. Compounds sharing the same scaffold were grouped into buckets, which were then sorted in descending order based on the number of compounds they contained. An 8:1:1 ratio was applied to split the data into training, validation, and test sets, ensuring that scaffolds did not overlap between sets.

In our initial experiments using random splitting, HelixADMET, particularly with the LiteGEM model, achieved comparable or higher performance than OmniMol on the metabolism category datasets. However, HelixADMET utilized significantly more data entries than ours across all datasets, especially in the substrates group (9,236

instances from five datasets in HelixADMET vs. 3,344 instances from ADMETLab 2.0 in our work). To ensure a fair comparison under similar conditions, we conducted experiments by jointly training on the metabolism datasets using our method with scaffold splitting and compared our results with those reported by HelixADMET.

The results of this comparison are presented in Supplementary Table 16.

Supplementary Table 16 Comparison of model performance on metabolism datasets using scaffold split and HelixADMET datasets. The table reports the AUC-ROC scores for OmniMol and HelixADMET (LiteGEM model). The best performance for each task is highlighted in bold.

Task Name	HelixADMET Mean	HelixADMET STD	OmniMol Mean	OmniMol STD
CYP1A2-inh	0.916	0.003	**0.942**	0.013
CYP1A2-sub	**0.782**	0.013	0.738	0.055
CYP2C19-inh	0.886	0.004	**0.921**	0.004
CYP2C19-sub	0.770	0.021	**0.794**	0.066
CYP2C9-inh	0.896	0.004	**0.907**	0.003
CYP2C9-sub	**0.766**	0.017	0.695	0.041
CYP2D6-inh	0.895	0.004	**0.896**	0.010
CYP2D6-sub	0.765	0.021	**0.853**	0.047
CYP3A4-inh	**0.911**	0.008	0.890	0.016
CYP3A4-sub	0.738	0.028	**0.850**	0.034
Mean	0.833	0.012	**0.849**	0.028

From Supplementary Table 16, we observe that our method achieves superior performance on 7 out of 10 substrate datasets. Notably, we found that OmniMol achieved higher performance on most of the inhibitors datasets, **red**while achieved a large increase of performance on the substrates dataset, such as the AUC-ROC improvements up to 11.2% over HelixADMET on CYP3A4-sub dataset. This strengthen our arguments that with more samples available, our method can provide better result.

It is also important to note that our experiments were conducted using a subset of the data used by HelixADMET. While HelixADMET utilized over 40 datasets across different ADMET groups, amounting to 663,004 instances (including the PCBA dataset) and 225,075 instances (excluding PCBA), our comparative experiment used only 10 datasets from the metabolism category. Despite the smaller dataset size, our method demonstrated competitive or superior performance.

Based on scaling laws and previous experimental results, models generally achieve better performance with more available data. Therefore, we anticipate that the performance of our method could be further enhanced when trained on larger datasets, similar to those used by HelixADMET. Our findings suggest that OmniMol has strong potential and can achieve better performance than existing methods like HelixADMET, even with less data.

In summary, these results demonstrate that our method achieves competitive performance on metabolism-related ADMET prediction tasks using scaffold splitting and outperforms HelixADMET on substrate prediction tasks. This confirms the effectiveness of OmniMol in learning transferable representations across multiple ADMET tasks and its potential for improved performance with larger datasets.

18 Comparative Analysis of Atomic Attention Patterns Across ADMET Endpoints

To facilitate a more comprehensive investigation, we provide a comprehensive examination of OmniMol's atom-wise attention distributions compared to specialized models across eight ADMET-P endpoints. Our analysis demonstrates OmniMol's ability to autonomously identify chemically relevant structural features that align with established pharmaceutical knowledge:

- hERG Channel Inhibition: hERG liability remains a critical safety concern in drug development, as channel inhibition can lead to cardiac arrhythmias and QT prolongation [9]. OmniMol demonstrates remarkable precision in identifying structural features known to influence hERG binding, including basic centers, lipophilic regions, and aromatic moieties. The model's attention patterns show strong correlation with specialized predictors, particularly BayeshERG [18] (high similarity), while offering more nuanced interpretations than Pred-hERG [3] and HergSPred [39] (moderate to partial similarity).
- Mutagenicity (Ames): In assessing mutagenic potential, OmniMol effectively identifies established structural alerts including nitrogen mustards, nitro groups, aziridines, and azides [1, 25]. The attention distribution shows exceptional alignment with Walter et al.'s work [33], demonstrating high-fidelity detection of genotoxicity-related structural features.
- Human Hepatotoxicity (H-HT)/Drug Induced Liver Injury (DILI): Drug-induced liver injury represents a major concern in pharmaceutical development. OmniMol accurately highlights structural features associated with hepatotoxicity, including nitroso groups, nitrogen mustards, and hydrazines [37]. The model's attention patterns show strong concordance with VenomPred 2.0 [8], particularly in identifying hepatotoxic structural alerts.
- Rodent Acute Toxicity (ROA): For acute toxicity prediction, OmniMol demonstrates sophisticated recognition of toxicophores, particularly azo and phosphate ester groups [23]. The attention distribution exhibits remarkable similarity with VenomPred 2.0, validating the model's capability to identify acute toxicity determinants.
- Carcinogenicity: OmniMol effectively identifies established carcinogenic structural alerts, including azo groups, aromatic N-oxides, and phosphate esters [1, 25]. The attention patterns show moderate similarity with CarcGC [37], while providing additional granularity in feature identification.
- Eye Corrosion/Irritation (EC/EI): The model accurately highlights structural features known to cause ocular toxicity, including active bromine atoms, isocyanates, and groups affecting acid-base properties [7]. OmniMol's attention distribution is highly similar to VenomPred 2.0, demonstrating high-fidelity detection of ocular toxicophores.
- Plasma Protein Binding (PPB): In assessing protein binding potential, OmniMol adopts a comprehensive approach, distributing attention across multiple molecular regions to account for the complex physicochemical factors affecting PPB [29]. This holistic interpretation shows moderate similarity with IDL-PPBopt [22], reflecting the multifaceted nature of protein-ligand interactions.
- Skin Sensitization: OmniMol successfully identifies key structural features associated with dermal sensitization, including β-lactams, aldehydes, and carboxylic acids [11]. The attention patterns demonstrate moderate correlation with Pred-skin 3.0 [2], while providing enhanced resolution of sensitization-related structural elements.

This comprehensive analysis demonstrates OmniMol's sophisticated chemical reasoning capabilities across diverse ADMET-P endpoints. The model's attention distributions consistently align with established pharmaceutical knowledge while offering additional interpretative depth compared to specialized single-task models.

19 Comparative Validation of Structure-Activity Relationship Interpretations

Based on the functional group analysis, we provide detailed structure-activity relationship (SAR) study examples across four critical ADMET properties to validate the model's practical utility in molecular optimization, corresponding to Fig. 6. Our analysis demonstrates OmniMol's capability to identify and track structural modifications that influence molecular properties:

- hERG Channel Inhibition: OmniMol effectively identifies known structural features affecting hERG liability, including basic centers, lipophilic regions, and conformational elements [9]. In published optimization cases [27], the model's attention patterns accurately track modifications that reduce hERG risk, showing decreased attention values on altered functional groups. This aligns with established strategies such as basicity reduction and conformational modification.
- Oral Bioavailability: Consistent with Lipinski's Rule of Five principles, OmniMol's attention distribution highlights molecular features critical for oral absorption [27]. The model demonstrates particular sensitivity to modifications affecting molecular weight, hydrogen bonding capacity, and lipophilicity, showing reduced attention on optimized structural elements that improve bioavailability profiles.
- Blood-Brain Barrier Penetration (BBBP): The model accurately tracks key physicochemical properties governing BBBP, including lipophilicity, molecular polarity, and hydrogen bonding potential [10]. In documented optimization cases [13], attention patterns clearly reflect structural modifications that enhance or restrict brain penetration.
- P-glycoprotein Substrate Recognition: For P-glycoprotein substrate assessment [27], OmniMol exhibits remarkable sensitivity to subtle structural changes. The model's attention distributions accurately reflect how minor modifications in type and position of atoms affect substrate recognition [5], aligning with experimental MDCK-MDR1 assay results.

To further validate OmniMol's interpretability and performance, we conducted a comparative analysis focusing on hERG inhibition prediction against three specialized models (Supplementary Fig. 3). This study examined three representative optimization cases from published literature, comparing the attention distribution of all four models in typical SAR study examples.

OmniMol demonstrates superior performance in identifying therapeutically relevant structural modifications compared to specialized models. While BayeshERG, Pred-hERG, and HergSPred show varying degrees of success in detecting key functional groups, they often exhibit more rigid attention patterns that may not fully capture the nuanced structural changes in optimization scenarios. OmniMol's attention distributions more closely align with experimental assays, showing appropriate sensitivity to both major structural alterations and subtle atomic-level modifications [9]. This comparative analysis underscores OmniMol's potential as a more adaptable and precise tool for guiding molecular optimization in drug development.

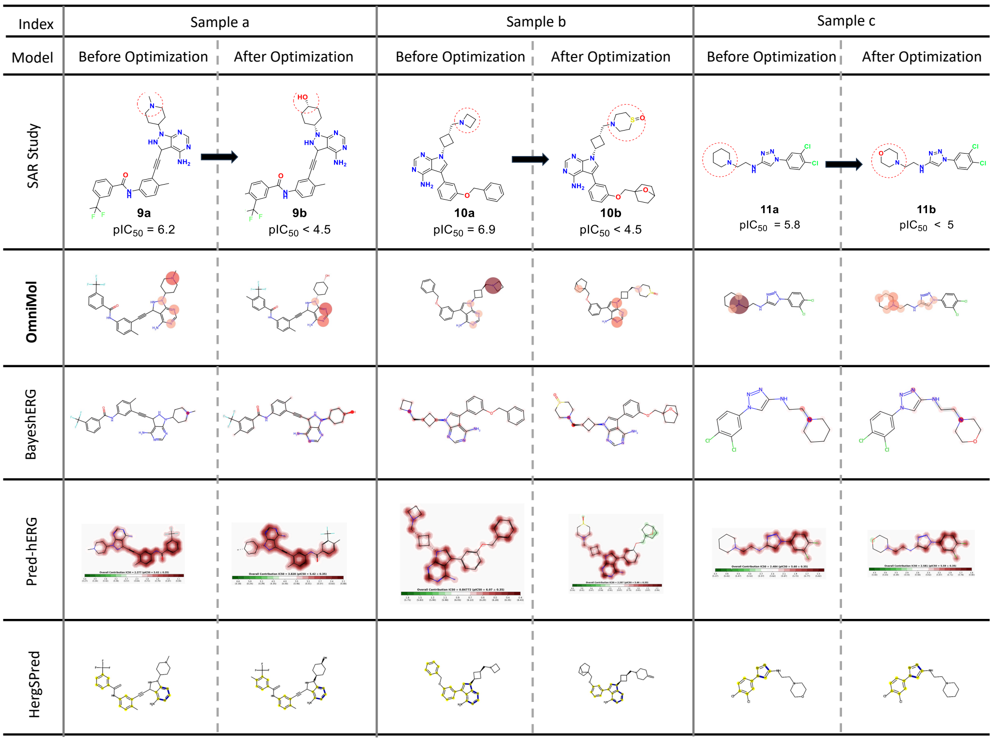

Supplementary Fig. 3 Comparative analysis of structural feature identification in hERG optimization cases. Row 1: Reference structures with experimentally validated modification sites. Rows 2-5: Feature importance distributions from OmniMol, BayeshERG, Pred-hERG, and HergSPred, respectively. Color intensity represents attention/importance weights. OmniMol demonstrates superior sensitivity to subtle structural modifications and better alignment with experimental optimization strategies compared to specialized models. Three representative cases show successful optimization through (A) basic center modification, (B) conformational adjustment, and (C) lipophilicity reduction.

20 Applicability Domain Analysis

The concept of the Applicability Domain (AD) is fundamental in developing robust and reliable Quantitative Structure-Activity Relationship (QSAR) models. According to Netzeva et al. [24], the AD delineates the chemical space where model predictions can be made with a defined level of confidence. An effective method that can determine whether a compound falls within the AD is especially important in practical applications. Predictions made outside applicability domain may lack accuracy, potentially undermining confidence in decisions regarding a compound's safety and therapeutic value.

In our initial AD implementation, we employed a simplistic range-based method utilizing seven fundamental molecular descriptors (Molecular Weight, LogP, Hydrogen Bond Donors, Hydrogen Bond Acceptors, Topological Polar Surface Area, Rotatable Bonds, and Ring Count). Compounds were classified into "In-domain", "Warning", and "Out-of-domain" categories based on descriptor percentiles derived from the training set. Although this approach provided a preliminary assessment of compound similarity, it lacked direct empirical validation linking these classifications to actual predictive reliability. Thus, there was a clear need to quantitatively demonstrate that predictions for "In-domain" compounds are indeed more accurate than those identified as "Out-of-domain".

Responding to this requirement, we have significantly enhanced our AD methodology by incorporating a broader and more comprehensive set of molecular descriptors and adopting the Isolation Forest algorithm [20]. This advanced approach enables more robust, data-driven AD definitions and directly quantifies their relationship with predictive performance.

Molecular Descriptors for AD Assessment

To construct a thorough representation of our chemical space, we expanded our descriptor set to include 20 diverse molecular descriptors, capturing physicochemical properties, topological and electronic characteristics, stereochemical features, complexity, aromaticity, and atomic composition:

- Basic Physicochemical Properties (7): Molecular Weight (MolWt), LogP, Hydrogen Bond Donors (HBD), Hydrogen Bond Acceptors (HBA), Topological Polar Surface Area (TPSA), Rotatable Bonds (RB), Ring Count (RC).
- Topological Descriptors (3): Bertz Complexity Index (BertzCT), Molecular Connectivity Index (Chi1), Normalized Connectivity Index (Chi1n).
- Electronic Descriptors (3): Electrotopological State Sum (EStateSum), Partial Charge Surface Area descriptors (PEOE_VSA1, PEOE_VSA2).
- Stereochemistry and Symmetry (1): Number of Chiral Centers.
- Complexity Descriptors (2): Fraction of sp^3 hybridized carbons (Fsp3), Quantitative Estimate of Drug-likeness (QED).
- Aromaticity Descriptors (2): Number of Aromatic Rings, Count of Aromatic Atoms.
- Functional Groups and Atomic Composition (2): Number of Heteroatoms, Heavy Atom Count.

All descriptors were computed for both training and test compounds, with Python implementation available at our public repository (https://github.com/bowenwang77/OmniMol/blob/master/check_applicability_domain.py).

AD Definition using Isolation Forest

The Isolation Forest algorithm [20] was employed to characterize the Applicability Domain (AD) for each predictive model. Isolation Forest is an unsupervised machine learning algorithm adept at anomaly detection. Its core principle is to isolate observations by randomly selecting a feature and then randomly selecting a split value between the maximum and minimum values of the selected feature. Since anomalous instances are typically "few and different," they are often isolated closer to the root of the decision trees within the forest, resulting in shorter average path lengths, or lower "anomaly scores." Conversely, "normal" instances, which form the dense core of the data distribution, typically require more partitions to be isolated and thus have longer average path lengths or higher scores.

In the context of AD, this algorithm helps to distinguish compounds that are "typical" and well-represented by the training set (In-Domain, ID) from those that are structurally dissimilar or "atypical" (potential Out-of-Domain, OOD) based on their representation in the 20-dimensional molecular descriptor space. The use of ensemble methods like Isolation Forest for AD assessment, particularly for identifying outliers in descriptor space, is a recognized approach in the literature for QSAR modeling [4, 28, 31].

The process for defining and utilizing the AD in this study was as follows:

1. Model Training and Anomaly Scoring: For each endpoint, an Isolation Forest model was trained using the 20 calculated molecular descriptors derived from the respective training set compounds. This model learns the distribution of the training data in the descriptor space. Subsequently, for every compound (in both training and test sets), the trained Isolation Forest model calculates an anomaly score, reflecting how similar or dissimilar it is to the bulk of the training data.

2. Defining a Threshold for Validation Purposes: To empirically validate the Isolation Forest-based AD approach, we established a specific threshold that partitioned the dataset such that approximately 75% of the training compounds were designated as In-Domain (ID), with scores indicating higher similarity to the training data core. The remaining 25% were considered as a reference group of more dissimilar, potentially Out-of-Domain (OOD) compounds.
3. AD Classification for Performance Comparison: Using this empirically determined threshold, test set compounds were then classified as either ID or OOD, where the predictive performance are compared.

It is important to emphasize that while this 75%/25% split served our validation objective, end-users can adjust the AD threshold flexibly, depending on their confidence requirements and risk tolerance. A higher threshold yields a more conservative AD, whereas a lower threshold expands it.

Validation of AD Effectiveness

A critical step in our revised AD analysis was to validate whether this ID/OOD classification effectively distinguishes between reliable and less reliable predictions. To achieve this, we compared the predictive performance of our models on the ID and OOD subsets of the test data for each endpoint.

Classification Tasks (39 endpoints):

- Average proportion of ID test samples: 73.18
- ID samples consistently showed superior predictive metrics compared to OOD samples:
  - Accuracy improvement (ID vs. OOD): +0.0324 (macro-average)
  - F1-score improvement (ID vs. OOD): +0.0297 (macro-average)
- Out of 39 endpoints, 27 endpoints exhibited higher performance for ID samples compared to OOD samples in accuracy and F1-score.

For example, the SR-ARE endpoint showed an accuracy improvement of 0.070 (ID: 0.912 vs. OOD: 0.842) and corresponding improvement in F1-score (0.066).

Regression Tasks (12 endpoints):

- Average proportion of ID test samples: 73.45%.
- ID samples consistently showed significantly better predictive metrics compared to OOD samples:
  - MAE reduction (ID vs. OOD): -0.1489 (macro-average improvement)
  - R^2 increase (ID vs. OOD): +0.1064 (macro-average improvement)
- Out of 12 endpoints, 10 showed lower MAE and 8 showed higher R^2 for ID samples than OOD samples.

Detailed result is shown in Supplementary Fig. 4 and Supplementary Fig. 5.

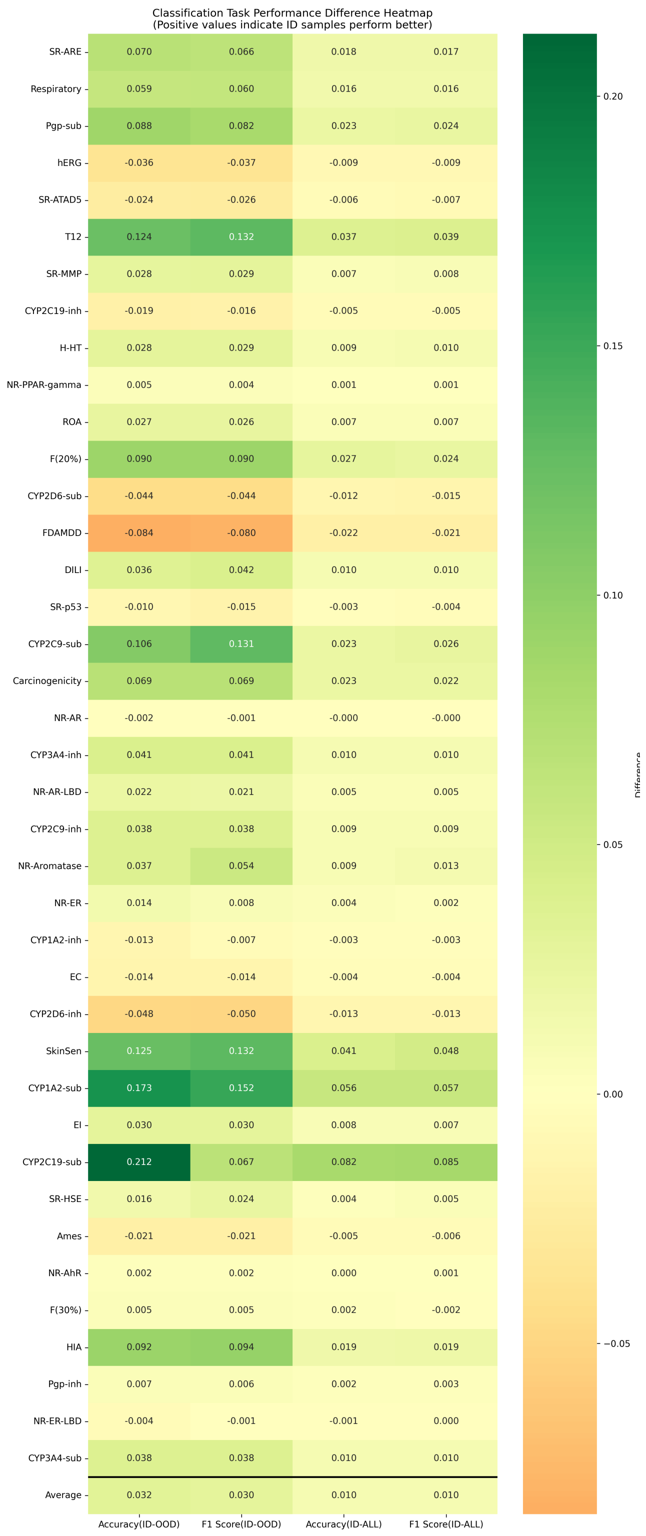

Supplementary Fig. 4 Classification task performace difference heatmap of AD

These results quantitatively confirm that compounds falling within the AD defined by our Isolation Forest approach are, on average, predicted with significantly higher

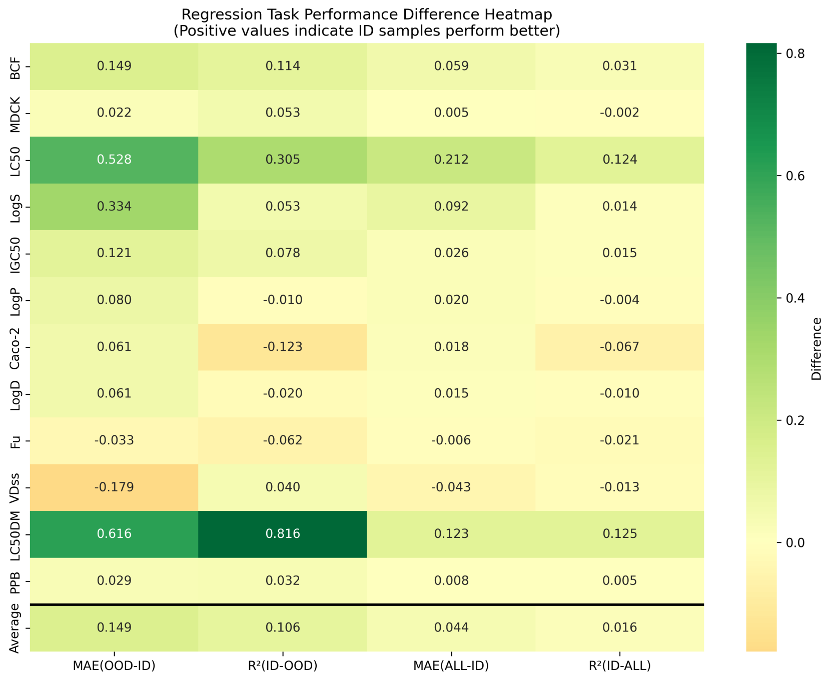

Supplementary Fig. 5 Regression task performace difference heatmap of AD.

accuracy (for classification) and lower error (for regression) than compounds classified as OOD. This directly addresses the concern about validating the AD's utility in identifying reliable predictions. By excluding compounds identified as OOD, we can achieve a more trustworthy set of predictions, thereby improving the practical applicability of our models. The analysis also indicates that our models primarily make misclassifications for compounds that are structurally more dissimilar to the training data (OOD), rather than solely due to proximity to decision boundaries among similar compounds.

In conclusion, the implemented AD framework, based on a comprehensive set of molecular descriptors and the Isolation Forest algorithm, effectively delineates the chemical space where our models exhibit higher predictive reliability. The empirical evidence showing superior performance for In-Domain compounds validates this approach and provides a valuable tool for assessing the trustworthiness of predictions.

21 Effectiveness of the Geometry-related Modules

To explicitly justify the effectiveness of our proposed geometry-aware modeling strategy, we conducted an additional targeted ablation study evaluating specific geometry-related modules.

Experimental Setting and Background. To ensure computational feasibility, we selected 8 representative ADMET-P subtasks (Ames, CYP1A2-inh, CYP3A4-inh, Carcinogenicity, DILI, F(20%), F(30%), and H-HT) from our full dataset. These tasks were specifically chosen due to their importance in our subsequent analysis presented in Figure 4, where we investigate task relationships and adaptive modeling strategies.

The computational complexity of this additional ablation was significantly reduced compared to our original full-scale model training. Specifically, the following hyperparameters were adjusted:

- Number of Transformer blocks: 2 (reduced from 4)
- Embedding dimension: 384 (reduced from 768)

- Feed-forward embedding dimension: 384 (reduced from 768)
- Total updates: 30,000 (reduced from 500,000)
- Warm-up updates: 1,000 (reduced from 5,000)

Detailed Explanation of Geometry-related Modules. Our geometry-aware modeling strategy is motivated by the philosophy that accurate molecular property prediction benefits when the model implicitly learns the underlying physics of molecular conformational relaxation, i.e., the transition from perturbed (high-energy) states to relaxed (equilibrium) states. To achieve this, we introduced three critical components explicitly evaluated in this study:

- Geometry Loss: Refers to supervising the model's predictions of geometries (atom positions and interatomic distances) during training. Specifically, we encourage the model to gradually converge from perturbed to equilibrium geometries by applying explicit loss functions on node-wise (atomic position) and edge-wise (interatomic distance) predictions.
- Geometry Noise Scale: Denotes the standard deviation of the Gaussian noise added to the equilibrium geometry at the training stage. A noise scale of 0 Å means no perturbation (original equilibrium geometry), 0.2 Å represents moderate perturbation reflecting physically plausible conformational fluctuations, and 20 Å represents extremely large random perturbation, significantly distorting atomic positions.
- Geometry Update: Indicates whether our model explicitly updates atomic positions iteratively during forward propagation. ✓ means the model incrementally predicts intermediate geometries, gradually approximating the relaxed equilibrium state. × means the model directly predicts final equilibrium geometry without iterative refinement.

Results and Detailed Discussion. The results of this additional ablation study are summarized in Supplementary Table 17.

Supplementary Table 17 Additional ablation study evaluating geometry-related modules on selected subtasks.

Experiment	Geometry Loss	Geometry Noise Scale (Å)	Geometry Update	Accuracy	ROC-AUC	Specificity	Sensitivity	MCC
A (Full Geometry Module)	✓	0.2	✓	**0.7644**	**0.8413**	**0.7909**	**0.7537**	**0.5320**
B	×	0.2	✓	0.7212	0.8099	0.7447	0.7212	0.4538
C	×	0	✓	0.7433	0.8292	0.7704	0.7322	0.4897
D	×	0	×	0.7354	0.8040	0.7451	0.7392	0.4713
E	✓	0.2	×	0.7188	0.7858	0.7177	0.7143	0.4238
F	×	20	✓	0.7002	0.7870	0.7318	0.6930	0.4159
G (Minimal Geometry Module)	×	20	×	0.7109	0.7811	0.7249	0.7095	0.4241

Key observations are as follows:

1. Importance of Iterative Geometry Updates (Compare A vs. E; C vs. D):
   - Comparing experiments A (full module) and E (no geometry update), we observe a substantial performance drop (ROC-AUC from 0.8413 to 0.7858) when iterative geometry updates are removed. This clearly highlights the crucial role of iterative prediction steps in accurately capturing the molecular relaxation process.
   - Intuitively, gradually predicting intermediate states (iterative updates) is essential, just like numerically solving differential equations requires incremental steps rather than a single jump.
2. Value of Geometry Supervision (Compare A vs. B; A vs. C):

- Removing geometry supervision (A vs. B) and removing geometry perturbation (A vs. C) both degrade performance significantly (ROC-AUC drops from 0.8413 in A to 0.8099 in B and 0.8292 in C).
- Geometry supervision helps the model explicitly learn representation towards physically realistic conformations. Without this supervision, the model must implicitly infer the relaxation process from noisy and distorted inputs, resulting in reduced accuracy.

3. Impact of Noise Magnitude (Compare G, F vs. A, B):

- Extremely large geometry perturbations (20 Å, experiments F and G) significantly distort structural information, leading to poor predictive performance regardless of geometry updates or supervision.
- This result aligns with expectation—adding excessively large noise essentially destroys meaningful geometric information, making subsequent geometry updates ineffective. Indeed, comparing experiments G (large perturbation without updates) and F (large perturbation with updates), we see minimal performance differences, confirming that iterative updates alone cannot rescue severely corrupted input.

4. Intrinsic Predictive Value of Original Geometry (Compare D vs. E; B vs. C):

- Comparing experiments D (original geometry, no updates, no supervision) and E (perturbed geometry, no updates, supervised), we observe that static original geometry delivers relatively strong baseline performance.
- This observation clearly indicates the intrinsic predictive value contained in the accurate equilibrium geometry itself. Conversely, adding noise without supervision (experiment B vs. C) notably reduces performance, as the model struggles to recover meaningful structural cues from corrupted geometry. Thus, precise molecular geometry inherently encodes valuable structural and relational information crucial for accurate property prediction.

5. Necessity of Combining Iterative Updates with Geometry Supervision (Compare G vs. E vs. A):

- Starting from the minimal setting (experiment G), adding only geometry supervision and moderate perturbation (experiment E) results in marginal improvement. However, adding iterative geometry updates on top of these components (experiment A) provides significant performance gains.
- Intuitively, this demonstrates that the relaxation process demands gradual, incremental geometric updates rather than single-step predictions. Just as incremental numerical methods (e.g., FEM simulations) require small iterative steps to accurately solve complex systems, our model benefits greatly from iterative geometry updates.

This additional ablation complements our previous ablation study (Supplementary Table 4), explicitly isolating and rigorously evaluating geometry-related modules. While the original analysis investigated broader architectural components (Meta-Encoder, SE(3)-Encoder, Mixed Training, Fine-tuning), this additional study explicitly demonstrates the standalone efficacy of geometry-aware modules. Collectively, these results provide robust, complementary empirical evidence supporting our proposed iterative geometry update, supervised geometry loss, and moderate perturbation strategy, clearly justifying the increased complexity of our modeling approach.

22 Reproducibility Example for LogS Prediction

In accordance with the journal's reproducibility guidelines for machine learning models, we provide a "twin model" example using a publicly available subset of our data. This example is designed to demonstrate the complete workflow and validate the performance of our OmniMol model on a representative task.

The experiment utilizes the training and testing split of the LogS (the logarithm of aqueous solubility value) subset of ADMETLab 2.0, which is included in the Source Data files. It includes 4320 molecules in total, with 3840 molecules for training and 480 molecules for testing. We initiated the training from the pre-trained checkpoint specified in Table Supplementary Table 8 (column E). The model was then fine-tuned for 20,000 steps using the hyperparameters detailed in column F of Table Supplementary Table 8.

The training process and test set results are illustrated in Figure Supplementary Fig. 6. The figure displays the training set loss, learning rate schedule, test set Mean Absolute Error (MAE), and test set Coefficient of Determination (R^2). Following this procedure, the fine-tuned model achieves a final MAE of 0.5092 and an R^2 of 0.8756 on the test set.

All artifacts required to replicate this experiment are publicly available. The LogS dataset is provided in the Source Data and our GitHub repository. The baseline pre-trained checkpoint and the final LogS-finetuned checkpoint are accessible from the same repository: https://github.com/bowenwang77/OmniMol. The complete raw data for the training curves are available in the Source Data file.

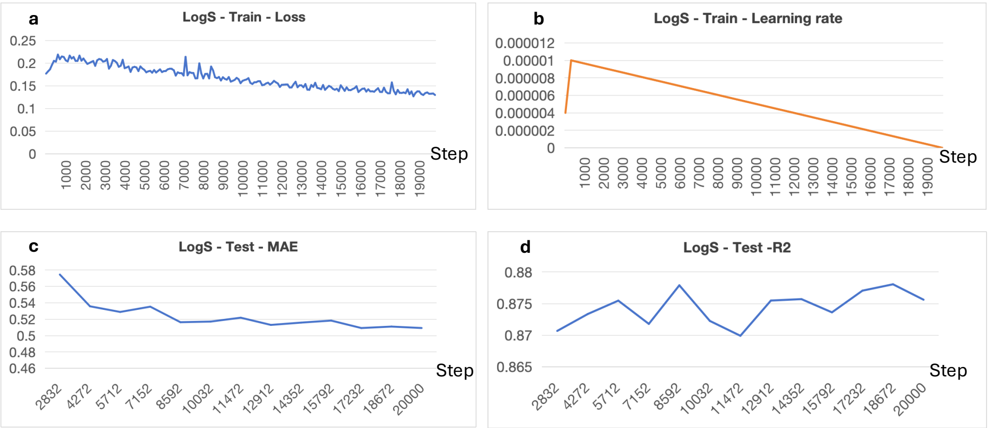

Supplementary Fig. 6 Training and validation of the OmniMol model on the LogS dataset. **a**, Training loss curve. **b**, Learning rate schedule over training steps. **c**, Mean Absolute Error (MAE) on the testing set. **d**, Coefficient of Determination (R^2) on the testing set.

References

[1] Romualdo Benigni and Cecilia Bossa. Structural alerts of mutagens and carcinogens. *Current Computer-Aided Drug Design*, 2(2):169–176, 2006.

[2] Joyce V B Borba, Rodolpho C Braga, Vinicius M Alves, Eugene N Muratov, Nicole Kleinstreuer, Alexander Tropsha, and Carolina Horta Andrade. Pred-skin: A web portal for accurate prediction of human skin sensitizers. *Chem. Res. Toxicol.*, 34(2):258–267, February 2021.

[3] Rodolpho C Braga, Vinicius M Alves, Meryck F B Silva, Eugene Muratov, Denis Fourches, Luciano M Lião, Alexander Tropsha, and Carolina H Andrade. Pred-hERG: A novel web-accessible computational tool for predicting cardiac toxicity. *Mol. Inform.*, 34(10):698–701, October 2015.

[4] Cosmin Alexandru Bugeac, Robert Ancuceanu, and Mihaela Dinu. Qsar models for active substances against pseudomonas aeruginosa using disk-diffusion test data. *Molecules*, 26(6):1734, 2021.

[5] Kevin C L Lam and Ganesh Rajaraman. Assessment of p-glycoprotein substrate and inhibition potential of test compounds in MDR1-transfected MDCK cells. *Curr. Protoc. Pharmacol.*, Chapter 7:Unit7.13, September 2012.

[6] Yaroslav Chushak, Jeffery M Gearhart, and Rebecca A Clewell. Structural alerts and machine learning modeling of "six-pack" toxicity as alternative to animal testing. *Computational Toxicology*, 27:100280, 2023.

[7] Peiwen Di, Mingyue Zheng, Tianbiao Yang, Geng Chen, Jianan Ren, Xutong Li, and Hualiang Jiang. Prediction of serious eye damage or eye irritation potential of compounds via consensus labelling models and active learning models based on uncertainty strategies. *Food and Chemical Toxicology*, 169:113420, 2022.

[8] Miriana Di Stefano, Salvatore Galati, Lisa Piazza, Carlotta Granchi, Simone Mancini, Filippo Fratini, Marco Macchia, Giulio Poli, and Tiziano Tuccinardi. VenomPred 2.0: A novel in silico platform for an extended and human interpretable toxicological profiling of small molecules. *J. Chem. Inf. Model.*, 64(7):2275–2289, April 2024.

[9] Amanda Garrido, Alban Lepailleur, Serge M Mignani, Patrick Dallemagne, and Christophe Rochais. herg toxicity assessment: Useful guidelines for drug design. *European journal of medicinal chemistry*, 195:112290, 2020.

[10] Werner J Geldenhuys, Afroz S Mohammad, Chris E Adkins, and Paul R Lockman. Molecular determinants of blood–brain barrier permeation. *Therapeutic delivery*, 6(8):961–971, 2015.

[11] Ingrid Gerner, Martin D Barratt, Stephan Zinke, Kerstin Schlegel, and Eva Schlede. Development and prevalidation of a list of structure–activity relationship rules to be used in expert systems for prediction of the skin-sensitising properties of chemicals. *Alternatives to Laboratory Animals*, 32(5):487–509, 2004.

[12] GH Hakimelahi and GA Khodarahmi. The identification of toxicophores for the prediction of mutagenicity, hepatotoxicity and cardiotoxicity. *Journal of the Iranian Chemical Society*, 2:244–267, 2005.

[13] Yu Hong, Yu Zhou, Jiang Wang, and Hong Liu. Lead compound optimization strategy (4)–improving blood-brain barrier permeability through structural modification. *Yao Xue Xue Bao*, 49(6):789–799, June 2014.

[14] Athar Husain, Vishal Makadia, Guru R Valicherla, Mohammed Riyazuddin, and Jiaur R Gayen. Approaches to minimize the effects of p-glycoprotein in drug transport: A review. *Drug Development Research*, 83(4):825–841, 2022.

[15] Xuelian Jia, Xia Wen, Daniel P Russo, Lauren M Aleksunes, and Hao Zhu. Mechanism-driven modeling of chemical hepatotoxicity using structural alerts and

an in vitro screening assay. *Journal of Hazardous Materials*, 436:129193, 2022.

[16] Amin Kamel and Shawn Harriman. Inhibition of cytochrome p450 enzymes and biochemical aspects of mechanism-based inactivation (mbi). *Drug Discovery Today: Technologies*, 10(1):e177–e189, 2013.

[17] Jeroen Kazius, Ross McGuire, and Roberta Bursi. Derivation and validation of toxicophores for mutagenicity prediction. *Journal of medicinal chemistry*, 48(1):312–320, 2005.

[18] Hyunho Kim, Minsu Park, Ingoo Lee, and Hojung Nam. BayeshERG: a robust, reliable and interpretable deep learning model for predicting hERG channel blockers. *Brief. Bioinform.*, 23(4), July 2022.

[19] Greg Landrum. Rdkit: Open-source cheminformatics software. 2016.

[20] Fei Tony Liu, Kai Ming Ting, and Zhi-Hua Zhou. Isolation forest. In *2008 eighth ieee international conference on data mining*, pages 413–422. IEEE, 2008.

[21] Edgar López-López and José L Medina-Franco. Towards decoding hepatotoxicity of approved drugs through navigation of multiverse and consensus chemical spaces. *Biomolecules*, 13(1):176, 2023.

[22] Chaofeng Lou, Hongbin Yang, Jiye Wang, Mengting Huang, Weihua Li, Guixia Liu, Philip W Lee, and Yun Tang. IDL-PPBopt: A strategy for prediction and optimization of human plasma protein binding of compounds via an interpretable deep learning method. *J. Chem. Inf. Model.*, 62(11):2788–2799, June 2022.

[23] Xi Luo, Tuan Xu, Deborah K Ngan, Menghang Xia, Jinghua Zhao, Srilatha Sakamuru, Anton Simeonov, and Ruili Huang. Prediction of chemical-induced acute toxicity using in vitro assay data and chemical structure. *Toxicol. Appl. Pharmacol.*, 492(117098):117098, September 2024.

[24] Tatiana I Netzeva, Andrew P Worth, Tom Aldenberg, Romualdo Benigni, Mark TD Cronin, Paola Gramatica, Joanna S Jaworska, Scott Kahn, Gilles Klopman, Carol A Marchant, et al. Current status of methods for defining the applicability domain of (quantitative) structure-activity relationships: The report and recommendations of ecvam workshop 52. *Alternatives to Laboratory Animals*, 33(2):155–173, 2005.

[25] Alja Plošnik, Marjan Vračko, and Marija Sollner Dolenc. Mutagenic and carcinogenic structural alerts and their mechanisms of action. *Arhiv za higijenu rada i toksikologiju*, 67(3):169–182, 2016.

[26] Nadine Schneider, Richard A Lewis, Nikolas Fechner, and Peter Ertl. Chiral cliffs: investigating the influence of chirality on binding affinity. *ChemMedChem*, 13(13):1315–1324, 2018.

[27] Patrick Schnider. The Medicinal Chemist's Guide to Solving ADMET Challenges. *The Royal Society of Chemistry*, 08 2021.

[28] Robert P Sheridan. Three useful dimensions for domain applicability in qsar models using random forest. *Journal of chemical information and modeling*, 52(3):814–823, 2012.

[29] Dennis A Smith, Li Di, and Edward H Kerns. The effect of plasma protein binding on in vivo efficacy: misconceptions in drug discovery. *Nat. Rev. Drug Discov.*, 9(12):929–939, December 2010.

[30] Clayton Springer and Katherine L Sokolnicki. A fingerprint pair analysis of herg inhibition data. *Chemistry Central Journal*, 7(1):1–8, 2013.

[31] Cindy Trinh, Silvia Lasala, Olivier Herbinet, and Dimitrios Meimaroglou. On the development of descriptor-based machine learning models for thermodynamic properties: Part 2—applicability domain and outliers. *Algorithms*, 16(12):573, 2023.

[32] Daniel F Veber, Stephen R Johnson, Hung-Yuan Cheng, Brian R Smith, Keith W Ward, and Kenneth D Kopple. Molecular properties that influence the oral bioavailability of drug candidates. *Journal of medicinal chemistry*, 45(12):2615–2623, 2002.

[33] Moritz Walter, Samuel J Webb, and Valerie J Gillet. Interpreting neural network models for toxicity prediction by extracting learned chemical features. *J. Chem. Inf. Model.*, 64(9):3670–3688, May 2024.

[34] Ning-Ning Wang, Chen Huang, Jie Dong, Zhi-Jiang Yao, Min-Feng Zhu, Zhen-Ke Deng, Ben Lv, Ai-Ping Lu, Alex F Chen, and Dong-Sheng Cao. Predicting human intestinal absorption with modified random forest approach: a comprehensive evaluation of molecular representation, unbalanced data, and applicability domain issues. *RSC advances*, 7(31):19007–19018, 2017.

[35] Qin Wang, Xiao Li, Hongbin Yang, Yingchun Cai, Yinyin Wang, Zhuang Wang, Weihua Li, Yun Tang, and Guixia Liu. In silico prediction of serious eye irritation or corrosion potential of chemicals. *RSC advances*, 7(11):6697–6703, 2017.

[36] Zhiyuan Wang, Piaopiao Zhao, Xiaoxiao Zhang, Xuan Xu, Weihua Li, Guixia Liu, and Yun Tang. In silico prediction of chemical respiratory toxicity via machine learning. *Computational Toxicology*, 18:100155, 2021.

[37] Huazhou Zhang, Hang Yi, Yuxing Hao, Lu Zhao, Wenxiao Pan, Qiao Xue, Xian Liu, Jianjie Fu, and Aiqian Zhang. Deciphering exogenous chemical carcinogenicity through interpretable deep learning: A novel approach for evaluating atmospheric pollutant hazards. *J. Hazard. Mater.*, 465(133092):133092, March 2024.

[38] Shanzhuo Zhang, Zhiyuan Yan, Yueyang Huang, Lihang Liu, Donglong He, Wei Wang, Xiaomin Fang, Xiaonan Zhang, Fan Wang, Hua Wu, et al. Helixadmet: a robust and endpoint extensible admet system incorporating self-supervised knowledge transfer. *Bioinformatics*, 38(13):3444–3453, 2022.

[39] Xudong Zhang, Jun Mao, Min Wei, Yifei Qi, and John ZH Zhang. Hergspred: Accurate classification of herg blockers/nonblockers with machine-learning models. *Journal of chemical information and modeling*, 62(8):1830–1839, 2022.
